# Cardiomyocyte-Specific RNF128 Attenuates Pathological Cardiac Hypertrophy Progression by Stabilizing SERCA2a through Lys63-Linked Polyubiquitination

**DOI:** 10.7150/thno.133221

**Published:** 2026-08-12

**Authors:** Yujie Zhang, Xuehan Liu, Liwen Yu, Changhao Liu, Jingwei Li, Qingmei Han, Xiaohong Wang, Lei Cao, Liangyu Cai, Linqi Jiao, Guohai Su, Meng Zhang, Cheng Zhang

**Affiliations:** 1 Key Laboratory of Cardiovascular Remodeling and Function Research, Shandong University Qilu Hospital, No. 107, Wenhua Xi Road, Jinan 250012, Shandong, China.; 2 Cardiovascular Disease Research Center of Shandong First Medical University, Central Hospital Affiliated to Shandong First Medical University, No. 105, Jiefang Road, Jinan 250013, China.; 3 Dongying People's Hospital, No. 317, Nanyi Road, Dongying, Shandong 257091, China.

**Keywords:** E3 ubiquitin ligase, cardiac hypertrophy, heart failure, autophagy, lysosomal degradation

## Abstract

Pathological cardiac hypertrophy is maladaptive cardiac remodeling induced by chronic adverse stimuli. In this study, the E3 ubiquitin ligase RNF128 was identified as a suppressor of pathological cardiac dysfunction with therapeutic value.

**Methods:**

The expression of Ring Finger protein 128 (RNF128) in pathological cardiac hypertrophy was characterized via public database analysis, scRNA-seq (single-cell RNA sequencing), and further validated in clinical myocardial samples and mouse disease models. The regulatory function of RNF128 in the progression of cardiac hypertrophy was verified *in vivo* by cardiomyocyte-specific RNF128 knockout mice and cTnT-AAV9-mediated RNF128 overexpression. Ang II (angiotensin II)-stimulated NMCMs (neonatal mouse cardiomyocytes) were used for* in vitro* validation. Moreover, the downstream target of RNF128 was identified through integrated analysis of scRNA-seq, interactome profiling and quantitative proteomics, followed by a panel of molecular assays.

**Results:**

Pathological cardiac hypertrophy reduced RNF128 expression in both human and murine samples. RNF128 deficiency aggravated cardiac dysfunction and pathological remodeling, while its overexpression protected cardiac function. Mechanistically, RNF128 directly interacted with SERCA2a, and catalyzed K63-linked polyubiquitination of SERCA2a at residue K476 (lysine 476), thereby impaired SERCA2a recognition by SQSTM1/p62. Consequently, RNF128 inhibited autophagy-lysosome-mediated degradation of SERCA2a.

**Conclusions:**

The findings of this study highlight RNF128 as a novel therapeutic target for heart failure, linking ubiquitination-dependent protein regulation to calcium handling in cardiomyocytes.

## Introduction

Hearts develop hypertrophic remodeling as an adaptive response against pathological stimuli. Sustained pathological stimuli drive the transition from myocardial hypertrophy to heart failure 1. Cardiomyocyte enlargement, heart dysfunction and interstitial fibrosis represent typical manifestations of pathological cardiac hypertrophy 2. Pathological signals exhibit multiple features including impaired calcium handling, reactivation of fetal gene programs, and fibrosis. Current treatments fail to reverse late-stage remodeling. Elucidating molecular mechanisms such as dysregulated calcium cycling is critical for developing targeted strategies to preserve myocardial integrity and delay heart failure progression.

SERCA2 (sarco/endoplasmic reticulum Ca²⁺-ATPase isoform 2) is the primary transporter responsible for Ca²⁺ reuptake in cardiomyocytes. SERCA2a is the major isoform in cardiomyocytes accounting for 97% of cytosolic calcium recycling 3. Its protein stability is vital for cardiomyocyte function. Downregulation of SERCA2a induces elevated calcium transient decay time constant (Ca^2+^*_τ_*) and reduced sarcoplasmic reticulum Ca^2+^ amplitude 4. It leads to impaired cardiomyocyte diastolic function 5, and triggers transcription of hypertrophic genes. Based on previous studies, SERCA2a can be degraded mainly through the proteasomal pathway 6, 7. The lysosomal pathway has also been reported recently 8. Autophagy-lysosome-mediated degradation is common for sarco/endoplasmic reticulum proteins 9. Modulating SERCA2a activity serves as a promising therapeutic intervention against hypertrophic cardiomyopathy and heart failure 10, 11.

Current studies reveal that SERCA2a has many types of post-translational modifications including acetylation 12, SUMOylation 7, 13 and ubiquitination 5, 14. Existing studies have demonstrated that K48-linked polyubiquitination of SERCA2a is tightly linked to its degradation via the proteasome pathway 5, 14. However, K63-linked polyubiquitination of SERCA2a remains poorly characterized. In past research, K63-linked ubiquitin moieties attached to SERCA2a at Lys628 can be eliminated by OTUD7B. Loss of the K63-linked chain at this residue enhances the interaction between SERCA2a and phospholamban (PLN), restricting SERCA2a-mediated Ca²⁺ handling, and driving cardiomyocyte hypertrophy 15. Additionally, gut-derived trimethylamine N-oxide (TMAO) has been reported to promote autophagy-lysosomal degradation of SERCA2a and modulate the progression of cardiac hypertrophy 8. In this study, we connected SERCA2a K63-linked polyubiquitination with autophagy-lysosome-mediated degradation.

RNF128 (Ring Finger Protein 128) is an E3 ubiquitin ligase initially identified in T cells, and has been shown to suppress inflammatory responses in immune cells 16-18. It has also been implicated in other diseases, such as cancer 19, non-alcoholic fatty liver disease 20 and atherosclerosis 21. RNF128 mediates ubiquitination of transmembrane proteins, primarily catalyzing K48-linked 22 and K63-linked 21 polyubiquitination. No prior studies have investigated its role in cardiac diseases. This study is the first to investigate the modulatory role of RNF128 in cardiomyocyte function and the pathogenesis of pathological cardiac hypertrophy. It exerts protective effects during myocardial hypertrophy and heart failure progression. This cardioprotective mechanism involves its catalytic mediation of ubiquitination modifications on SERCA2a. Furthermore, we discovered that RNF128 catalyzes SERCA2a’s K63-linked polyubiquitination, and suppresses its degradation through the autophagy-lysosomal pathway. These findings might provide a novel therapeutic target for future treatment.

## Materials and Methods

### Mice

We generated RNF128 cardiomyocyte-specific knockout (RNF128^cKO^) mice on the *C57BL/6J* background by crossing RNF128^flox/flox^ (RNF128^fl/fl^) mice with Myh6-Cre mouse lines. Both RNF128^fl/fl^ and Myh6-Cre strains were obtained from GemPharmatech (Nanjing, China). Age-matched littermate mice were selected for all subsequent experiments. All mice were maintained in an SPF-grade facility under a 12-h light/12-h dark cycle with free access to food and water.

Animal care and experimental procedures were reviewed and approved by the Animal Ethics Review Committee of the Experimental Animal Center, Qilu Hospital of Shandong University (SYXK 2023003; IACUC No. DWLL-2022-085). For genotype identification, we extracted nucleic acids from mouse tail tissue and performed genotyping PCR using primers targeting the Myh6-Cre sequence and RNF128^flox/flox^ allele.

To minimize variability related to sex hormones, only male mice were used.

### Animal experiments

Ang II cardiac hypertrophy model: 12 healthy male RNF128^cKO^ and 12 healthy male RNF128^fl/fl^ 8-week-old mice were treated with Ang II (1000 ng/kg/min) or normal saline for 4 weeks (6 mice were included in each group) via an osmotic pump (RWD), which was subcutaneously implanted on the dorsal region of each animal. Systolic blood pressure and body weight were measured at baseline and once a week during the 4-week intervention. The model was deemed successfully established when systolic blood pressure exceeded 150 mmHg and was sustained no less than 3 weeks. After 4 weeks of treatment, all mice were euthanized, and serum samples as well as heart tissues were harvested for subsequent analyses 5.

Transverse aortic constriction (TAC)-induced cardiac hypertrophy model: healthy male RNF128^cKO^ and RNF128^fl/fl^ 8-week-old mice (6 surviving mice were included in each group) were selected and anesthetized with tribromoethanol by intraperitoneal injection. After neck and chest depilation, the trachea was exposed via cervical incision, and the second rib was transected to open the thoracic cavity with a retractor. Following thymus separation, the aortic arch and left common carotid artery were exposed. The ascending aorta was ligated with a 6-0 suture alongside a 27G needle, which was removed afterward to generate standardized stenosis. Erythromycin ointment was applied to the incision to prevent infection and pneumothorax. Mice were euthanized 4 weeks post-surgery for sample collection, and animals that died perioperatively or postoperatively (mortality < 5%) were excluded.

### Human samples

Cardiac specimens were harvested from patients with pathological cardiac hypertrophy associated with valvular disease undergoing cardiac surgery and patients with end-stage heart failure due to dilated cardiomyopathy (DCM) undergoing left ventricular assist device implantation or heart transplantation. Non-cardiac disease control (NCC) myocardial samples were obtained from organ donors without cardiovascular disease. Written informed consent was acquired from all participants or their legal guardians prior to tissue collection. Clinical information and tissue specimens were anonymized to protect patient privacy. All collection procedures were performed at Qilu Hospital of Shandong University. This study was approved by the Ethics Committee of Qilu Hospital of Shandong University. Approval numbers were KYLL-202111-070 and KYLL-2019-569.

### Statistical analysis

All results reported in this article were obtained from no fewer than three independent biological replicates. Unless otherwise stated, values were presented as mean ± SD (standard deviation). The sample size “n” is provided for each assay. Unless noted, “n” represents biological replicates instead of technical replicates. Statistical analyses were performed using GraphPad Prism version 10.1.2. Normality of each dataset was assessed using the Shapiro–Wilk test, while homogeneity of variances was verified via *F*-test and Brown–Forsythe test. A p-value greater than 0.05 for the Shapiro–Wilk test indicated no significant deviation from a normal distribution; p > 0.05 for variance tests suggested equal variances between groups. For comparisons between two groups, unpaired Student’s t-test was applied when variances were equal, whereas Welch’s t-test was used when variances were unequal. Multiple comparisons of single-factor experimental groups were conducted using one-way ANOVA followed by Tukey’s or Dunnett’s multiple comparisons test for normally distributed data, or Kruskal-Wallis test followed by Dunn’s multiple comparisons test for non-normal datasets. Two-factor datasets were analyzed with two-way ANOVA followed by Šídák’s multiple comparisons test for parametric data. For non-parametric datasets, targeted Mann-Whitney U tests were performed only for pre-planned comparisons between two genotypes within the same experimental condition. Significance thresholds were defined as: ns, p > 0.05, * p < 0.05, ** p < 0.01, *** p < 0.001, **** p < 0.0001.

### Blinding and randomization strategies adopted in animal experiments

Sample size was estimated through pre-study statistical power analysis using G*Power version 3.1.9. Left ventricular ejection fraction (LVEF) was adopted as the primary endpoint for this statistical computation. The threshold of two-sided α error was defined as 0.05 with 80% statistical power (1-β). The standardized effect size (Cohen’s d = 1.6) was estimated based on our preliminary experimental raw data. Power analysis for two-sided independent-samples t-test with equal group allocation revealed a minimum sample size of 6 mice per experimental group.

All procedures and analyses adhered to strict single-blind assessment protocols. Two independent researchers responsible for echocardiography acquisition, histological staining quantification, and molecular data analysis were blinded to treatment conditions and group identities throughout the study. Grouping information was kept by a third researcher not involved in data collection and was only decoded after all statistical analyses were completed.

### Single-cell RNA sequencing

Cell suspensions were processed with the 10× Genomics Chromium Single Cell 3′ v3 Kit according to the manufacturer’s instructions, with ~10,000 nuclei captured from each sample on the 10× Chromium platform. cDNA amplification and library construction were performed following standard protocols. Library sequencing was performed using the Illumina NovaSeq 6000 system (150 bp paired-end multiplexed mode) at Analytical Biosciences (Beijing, China). Quality control screening removed nuclei with mitochondrial read proportions exceeding 5% or UMI counts outside 200 to 6000. Doublets were filtered using the DoubletFinder R package. After excluding low-quality reads and doublets, these nuclei were annotated using classical cell type markers, including fibroblasts (*Dcn*,* Serpinf1*), endothelial cells (*Pecam1*, *Cdh5*), cardiomyocytes (*Tnnt2*, *Myh7*), myeloid cells (*C1qa*, *Cd68*), pericytes (*Rgs5*, *Nrxn1*), smooth muscle cells (*Myh11*, *Tagln*), lymphoid cells (*Cd3e*, *Cd8a*), neurons (*Gpm6b*, *S100b*), mast cells (*Kit*, *Cpa3*), adipocytes (*Adipoq*, *Gpam*).

Single-cell RNA-seq data for 3 RNF128^fl/fl^ mice and 3 RNF128^cKO^ mice: Fresh heart tissues were dissected and frozen in liquid nitrogen immediately. Samples were stored at -80 °C until nuclei isolation. Nuclei were extracted using a commercial nuclei-isolation kit and standard Dounce homogenization method, filtered through a 40 μm cell strainer, and stained with trypan blue to assess integrity and concentration. Only nuclei preparations with > 90% viability were used for library construction.

All analyses were performed using the R package Seurat v5.2.1. Briefly, quality control was conducted to filter low-quality cells with 200-6000 detected genes, and mitochondrial gene expression ratio < 5%. After normalization with LogNormalize and selection of 5000 highly variable genes, batch effects were integrated using the Harmony method. For dimensionality reduction and visualization, the Uniform Manifold Approximation and Projection (UMAP) method was applied to the clustering results. UMAP preserves the topological relationships between cells through nonlinear dimensionality reduction, allowing the identified cell clusters to be visualized in two-dimensional space. Cluster-specific marker genes were identified using the FindAllMarkers function. Differentially expressed genes (DEGs) between groups were analyzed using the FindMarkers function, with thresholds set at |log2foldchange| > 0.15 and an adjusted p-value < 0.05. Functional enrichment analyses of GO and KEGG were conducted using the clusterProfiler package (v4.14.6). GSEA was performed using the clusterProfiler with the MSigDB Hallmark gene set collection.

### LC-MS/MS Analysis

Immunoprecipitation experiments were performed by adding RNF128 antibody to NMCM lysate samples, with IgG set as the negative control. LC-MS/MS detection was commissioned to PTM Bio Co., Ltd. (Zhejiang, China). Substrate proteins capable of binding to RNF128 were ultimately filtered according to the matching scores and relative quantity of the identified proteins.

Samples were homogenized in liquid nitrogen, lysed with SDT buffer, boiled for 3 min, and sonicated for 2 min. Supernatants were collected after spinning at 16,000 g for 20 min. At 4 °C, protein content was quantified via the BCA assay kit. Peptide separation was performed on a Vanquish Neo UHPLC system (Thermo Scientific). DIA-MS data were processed with DIA-NN for database searching and protein quantification against the UniProtKB *Mus musculus* (TaxID 10090) database. For differential abundance analysis of the quantified ubiquitination sites, we first ensured that each identified site retained at least three non-zero quantitative values in at least one experimental group. Zero values in the quantitative matrix were imputed using the k-nearest neighbor (KNN) algorithm, followed by pairwise comparisons between groups. Sites with |log2foldchange| > 1, raw p-value < 0.05 and adjusted Q-value ≤ 0.01 in pairwise comparisons were identified as significantly differentially modified sites. All bioinformatic analyses were performed using Microsoft Excel and R software.

Remaining methods and materials are listed in the Supplementary materials document.

## Results

### RNF128 is downregulated in pathological cardiac hypertrophy

**Figure 1:**
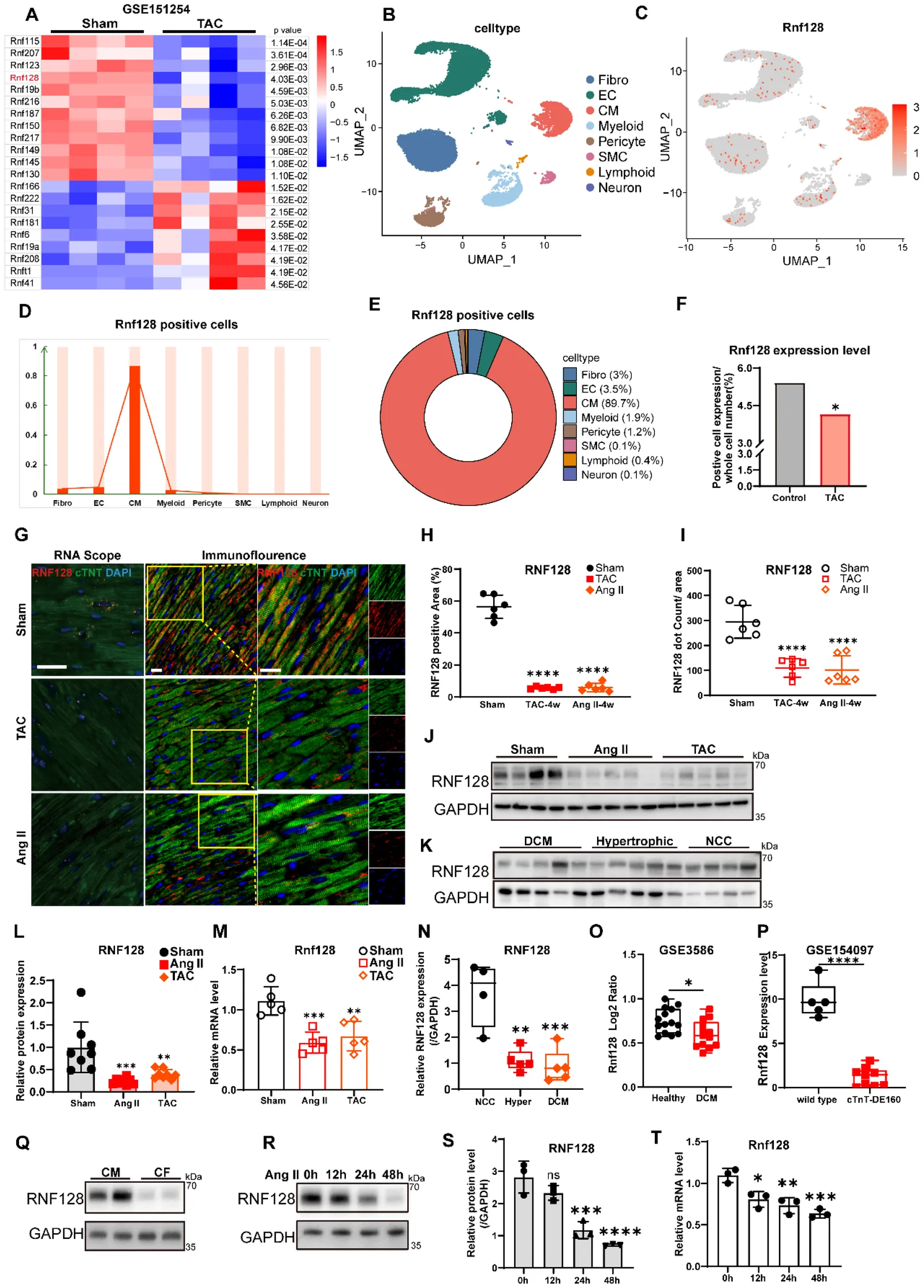
** RNF128 is downregulated in cardiomyocytes during cardiac hypertrophy. A**, Heatmap of differentially expressed RNF family members in GSE151254 datasets. **B**, single-cell RNA sequencing (scRNA-seq) showing 8 clusters of cells from mouse hearts. **C** to **E**, RNF128 is highly enriched in cardiomyocytes.** F**, scRNA-seq revealed a reduced proportion of total positive expression abundance of Rnf128 among total cardiac cells after TAC surgery.** G**, Representative images of in situ RNAscope and immunofluorescence staining of RNF128 in sham and hypertrophic heart sections (scale bar = 50 μm). **H**, The percentage of RNF128 positive area in sham and hypertrophic heart (n = 6 mice, vs sham, one-way ANOVA). **I**, Quantification of RNF128 RNA dots in sham and hypertrophic heart tissue (n = 6 mice per group, vs sham, one-way ANOVA). **J**, Representative immunoblotting images of RNF128 protein levels in sham, Ang II and TAC tissue. **K**, Representative western blot images showing RNF128 level in dilated cardiomyopathy patients (DCM, n = 5), hypertrophic patients (n = 5) and non-cardiac disease control donors (NCC, n = 4). **L** to** M**, Quantification of RNF128 level in mouse tissues (n ≥ 5 mice, vs sham, one-way ANOVA). **N**, RNF128 protein expression in human samples (n ≥ 4, one-way ANOVA).** O**, Human heart tissues RNA datasets from GSE3586. **P**, RNA datasets from GSE154097. **Q**, RNF128 is mainly expressed in cardiomyocytes but not fibroblasts *in vitro*.** R** to** T**, Cardiomyocyte RNF128 is suppressed by Ang II *in vitro* (n = 3 biological replicates, vs 0 h, one-way ANOVA). (ns, p > 0.05, * p < 0.05, ** p < 0.01, *** p < 0.001, **** p < 0.0001).

We analyzed murine cardiac mRNA profiles from the GSE151254 dataset and revealed significant dysregulation in multiple RING finger (RNF) family members following transverse aortic constriction (TAC) (Figure 1A). Cardiac cells were harvested from male *C57BL/6J* mice that underwent sham or TAC surgery. Cells from each group were pooled into a single sample and subjected to scRNA-seq (single cell RNA sequencing) for full-scale single-cell transcriptomic profiles. Cells were defined into 8 distinct subpopulations (Figure 1B). Among the top four RNF family members ranked by p-value in GSE151254, scRNA-seq analysis demonstrated cardiomyocyte-specific enrichment of *Rnf207* and *Rnf128* (Figure 1C-E, Figure S1B). *Rnf123* and *Rnf115* exhibited ubiquitous expression patterns (Figure S1B). RNF207 has been reported to participate in the progression of pathological cardiac hypertrophy 23, therefore we focused on the biological function of *Rnf128*. *Rnf128* expression was downregulated in TAC hearts (Figure 1F). Immunofluorescence and RNA Scope in situ hybridization confirmed reduced RNF128 protein and mRNA levels in both TAC and Ang II (angiotensin II) models (Figure 1G-I, Figure S1A). Cardiac hypertrophy models induced by TAC surgery or angiotensin II (Ang II) stimulation were established, followed by the detection of RNF128 transcript and protein abundance in heart tissues (Figure 1J, 1L-M). To verify the potential involvement of RNF128 in human pathological conditions, we further assessed the RNF128 protein level in human clinical specimens. Human myocardial samples were collected from three cohorts: non-cardiac disease control (NCC) donors, patients with pathological cardiac hypertrophy associated with valvular heart disease (Hypertrophic), and patients with dilated cardiomyopathy (DCM) (Figure 1K, 1N). Analysis of dataset GSE3586 from public database Gene Expression Omnibus (GEO) also showed similar downregulation in dilated cardiomyopathy patients. Analysis of the GSE154097 dataset showed that human embryonic stem cells (hESCs) carrying the hypertrophic cardiomyopathy-associated cardiac troponin T (cTnT) DE160 mutation exhibited reduced *Rnf128* expression compared with control hESCs (Figure 1O-P). Primary neonatal mouse cardiomyocytes (NMCMs) and fibroblasts (NMCFs) were isolated for *in vitro* validation, revealing predominant RNF128 expression in cardiomyocytes (Figure 1Q). In cardiomyocytes, RNF128 expression showed a progressive, time-dependent decrease upon Ang II stimulation (Figure 1R-T).

### Cardiomyocyte RNF128 deficiency promotes TAC-mediated cardiac hypertrophy and dysfunction

**Figure 2:**
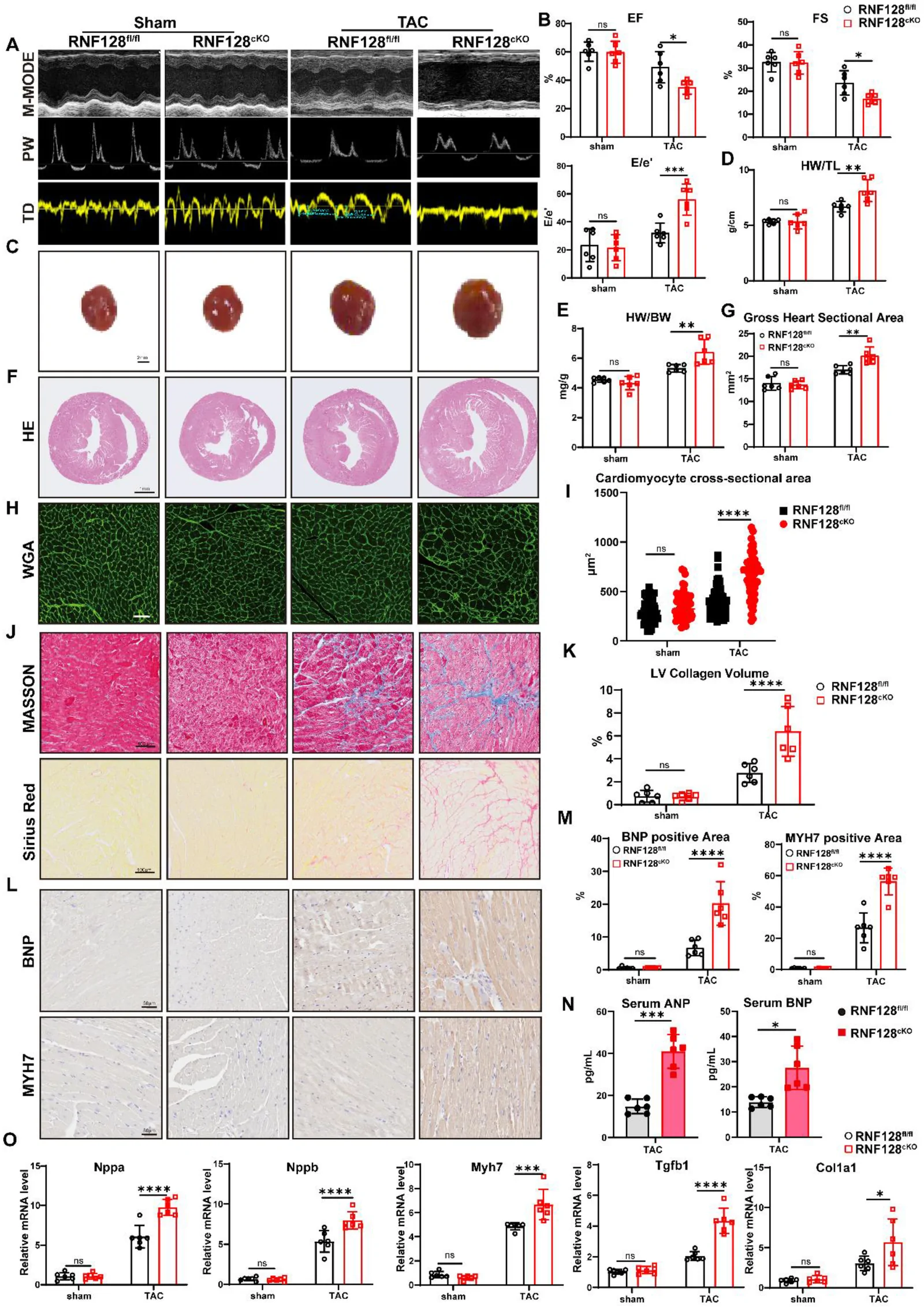
** RNF128 deficiency aggravates TAC (transverse aortic constriction) induced cardiac hypertrophy. A**, Representative M-mode, pulse-wave doppler (PW) and tissue doppler (TD) images from each group in mice. **B**, Ejection fraction (EF), fractional shortening (FS) and early diastolic mitral inflow velocity to early diastolic mitral annular velocity ratio (E/e’). **C**, Representative image of whole heart (scale bar = 2 mm). **D**, Heart weight to tibial length ratio (HW/TL). **E**, Heart weight to body weight ratio (HW/BW). **F**, Representative image of hematoxylin-eosin staining (H&E). **G**, Gross heart sectional area is elevated in TAC treated RNF128^cKO^ group. **H**, Wheat germ agglutinin (WGA) staining showing cardiomyocyte cross-sectional area. **I**, Quantification of cardiomyocyte cross-sectional area measured by WGA (n = 60 cells from 6 mice of each group, Kruskal–Wallis H test, and post-hoc pairwise comparisons were conducted with the Mann–Whitney U test). **J**, Masson and Sirius Red staining in heart sections. **K**, Statistical analysis of left ventricular fibrosis by Sirius Red staining.** L** to** M**, Immunohistochemical Staining of BNP and MYH7 in heart sections. **N**, The serum ANP and BNP concentrations in TAC models (n = 6 per group, Welch’s t-test). **O**, Real-time qPCR analysis of *Nppa*, *Nppb*, *MYH7*, *Tgfb1* and *Col1a1* in heart tissues. (**B, D, E, G, K, M, O**: n = 6 mice per group. “Bar-graph data are shown as mean ± SD. Šídák’s multiple comparisons test was performed after two-way ANOVA for comparisons between genotypes within each treatment condition. Corrected p-values annotated accordingly. Relative mRNA transcript abundance was normalized to sham RNF128^fl/fl^ mice, whose expression value was defined as 1. ns p > 0.05, * p < 0.05, ** p < 0.01, *** p < 0.001, **** p < 0.0001.)

RNF128^cKO^ mice (cardiomyocyte-specific RNF128 knockout) were generated by crossbreeding Myh6-Cre mice with RNF128^flox/flox^ (RNF128^fl/fl^) mice. Total RNA and protein samples were extracted from heart and other tissues to verify the specific knockout of RNF128 in myocardial tissue and quantify its knockout efficiency (Figure S2A-B). Eight-week-old mice were subjected to TAC surgery (Figure S2C). Echocardiographic analysis revealed that RNF128^cKO^ mice exhibited significantly impaired systolic function after TAC compared to RNF128^fl/fl^ littermates, indicated by markedly reduced EF (ejection fraction) and FS (fractional shortening). Furthermore, RNF128^cKO^ hearts showed compromised diastolic function, evidenced by a significantly increased E/e' (ratio of early diastolic mitral inflow velocity to early diastolic mitral annular velocity) (Figure 2A-B). Cardiac remodeling parameters were also exacerbated in RNF128^cKO^ mice. Hearts displayed hypertrophic remodeling characterized by increased LVPW (left ventricular posterior wall) thickness (Table S1) and substantial cardiac enlargement compared to RNF128^fl/fl^ controls (Figure 2C). RNF128^cKO^ mice exhibited elevated HW/BW (heart weight-to-body weight ratio) and HW/TL (heart weight-to-tibia length ratio) (Figure 2D-E). Whole-heart cross-sectional area was enlarged in RNF128^cKO^ mice (Figure 2F-G). Additional histological examination demonstrated markedly enlarged cardiomyocyte cross-sectional areas (Figure 2H-I) as well as aggravated interstitial myocardial fibrosis in RNF128^cKO^ mouse hearts (Figure 2J, K). IHC (Immunohistochemical) staining results further verified that the myocardial expression of hypertrophy markers BNP and MYH7 was significantly upregulated in RNF128^cKO^ cardiac tissues (Figure 2L-M). Using enzyme-linked immunosorbent assay (ELISA), we detected significantly higher serum concentrations of atrial natriuretic peptide (ANP) and brain natriuretic peptide (BNP) in RNF128^cKO^ mice (Figure 2N). Consistent with this observation, quantitative real-time PCR (qRT-PCR) showed that the mRNA levels of cardiac remodeling markers (*Nppa*, *Nppb*, *Myh7*, *Col1a1*, *Tgfb1*) were markedly increased in RNF128^cKO^ myocardium (Figure 2O).

### Cardiac RNF128 knockout exacerbates Angiotensin II-induced cardiac remodeling

**Figure 3:**
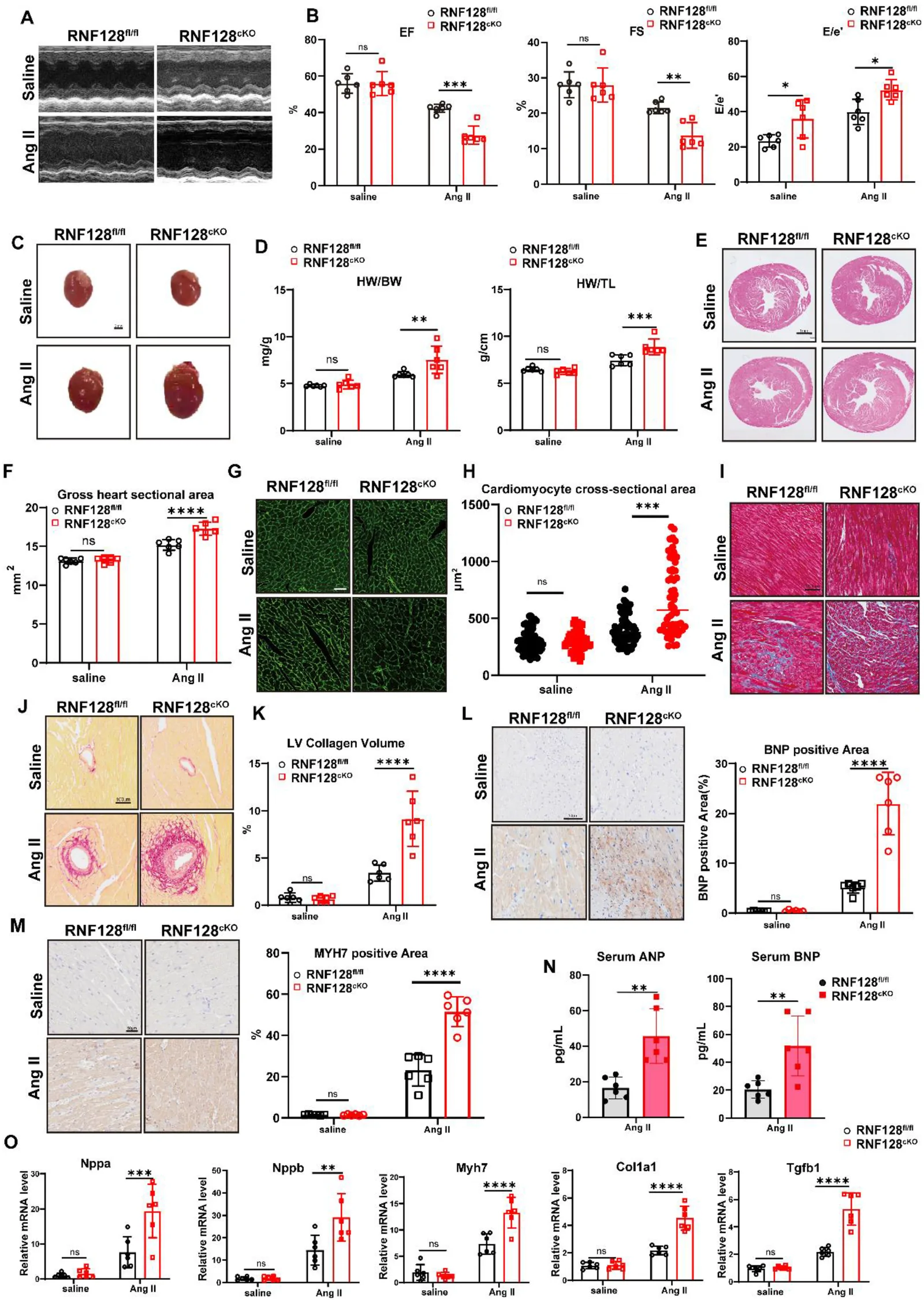
** RNF128 knockout promotes angiotensin II (Ang II)-induced myocardial hypertrophy. A**, Representative M-mode image from each group. **B**, Cardiac function is significantly impaired in RNF128^cKO^ mice. **C**, Representative gross-heart images (scale bar = 2 mm). **D**, RNF128^cKO^ mice show exacerbated cardiac hypertrophy, indicated by elevated HW/BW and HW/TL ratios. **E**, representative H&E-stained sections. **F**, quantification of cardiac cross-sectional area. **G** to** H**, WGA staining shows significantly enlarged cross-sectional area of single cardiomyocytes in RNF128^cKO^ mice. (scale bar = 50 μm, n = 60 cells from 6 mice, Kruskal–Wallis H test, and post-hoc pairwise comparisons were conducted with the Mann–Whitney U test). **I** to** K**, Masson and Sirius Red staining revealed higher left ventricular fibrosis in RNF128^cKO^ mice. **L**, Immunohistochemical staining of BNP in heart sections. **M**, Immunohistochemical staining of MYH7 in heart sections. **N**, The serum ANP and BNP concentration. (n = 6 mice per group, Student’s t-test). **O**, Real-time qPCR analysis of *Nppa*, *Nppb*, *MYH7*, *Tgfb1* and *Col1a1* in heart tissues. (**B**, **D**, **F**,** H**,** K**,** L**,** M**,** O**: n = 6 mice per group. All histogram data are displayed as mean ± standard deviation. Šídák’s multiple comparisons test was performed after two-way ANOVA for RNF128^fl/fl^ and RNF128^cKO^ within saline or Ang II group. Corrected p-values annotated accordingly. Relative mRNA transcript abundance was normalized to saline RNF128^fl/fl^ mice, whose expression value was defined as 1. ns p > 0.05, * p < 0.05, ** p < 0.01, *** p < 0.001, **** p < 0.0001.)

To further explore the regulatory role of RNF128 in cardiac remodeling, we administered 1000 ng/kg/min Ang II infusion to RNF128^fl/fl^ and RNF128^cKO^ mice by osmotic minipumps for 4 weeks (Figure S3A). Both RNF128^cKO^ and their RNF128^fl/fl^ littermates exhibited significant blood pressure elevation post-infusion. There were no baseline differences in blood pressure between genotypes (Figure S3B). Echocardiographic assessment revealed exacerbated cardiac systolic and diastolic dysfunction in RNF128^cKO^ mice (Figure 3A-B, Figure S3C). RNF128^cKO^ mice displayed cardiac enlargement (Table S2) and elevated heart weight ratio (Figure 3C-D). H&E staining showed expanded gross cardiac cross-sectional areas (Figure 3E-F), and WGA revealed larger cardiomyocyte cross-sectional area (Figure 3G-H). Masson and Sirius red staining analyses demonstrated enhanced myocardial fibrosis in RNF128^cKO^ mice (Figure 3I-K). IHC showed increased myocardial BNP and MYH7 expression in RNF128^cKO^ sections (Figure 3L-M). Serum ANP and BNP levels (ELISA) were increased in RNF128^cKO^ mice (Figure 3N). Cardiac remodeling markers (*Nppa*, *Nppb*, *Myh7*, *Col1a1*, *Tgfb1*) were elevated at the mRNA level (Figure 3O).

### RNF128 affects cardiomyocyte hypertrophy and intracellular Ca^2+^ restoration

**Figure 4:**
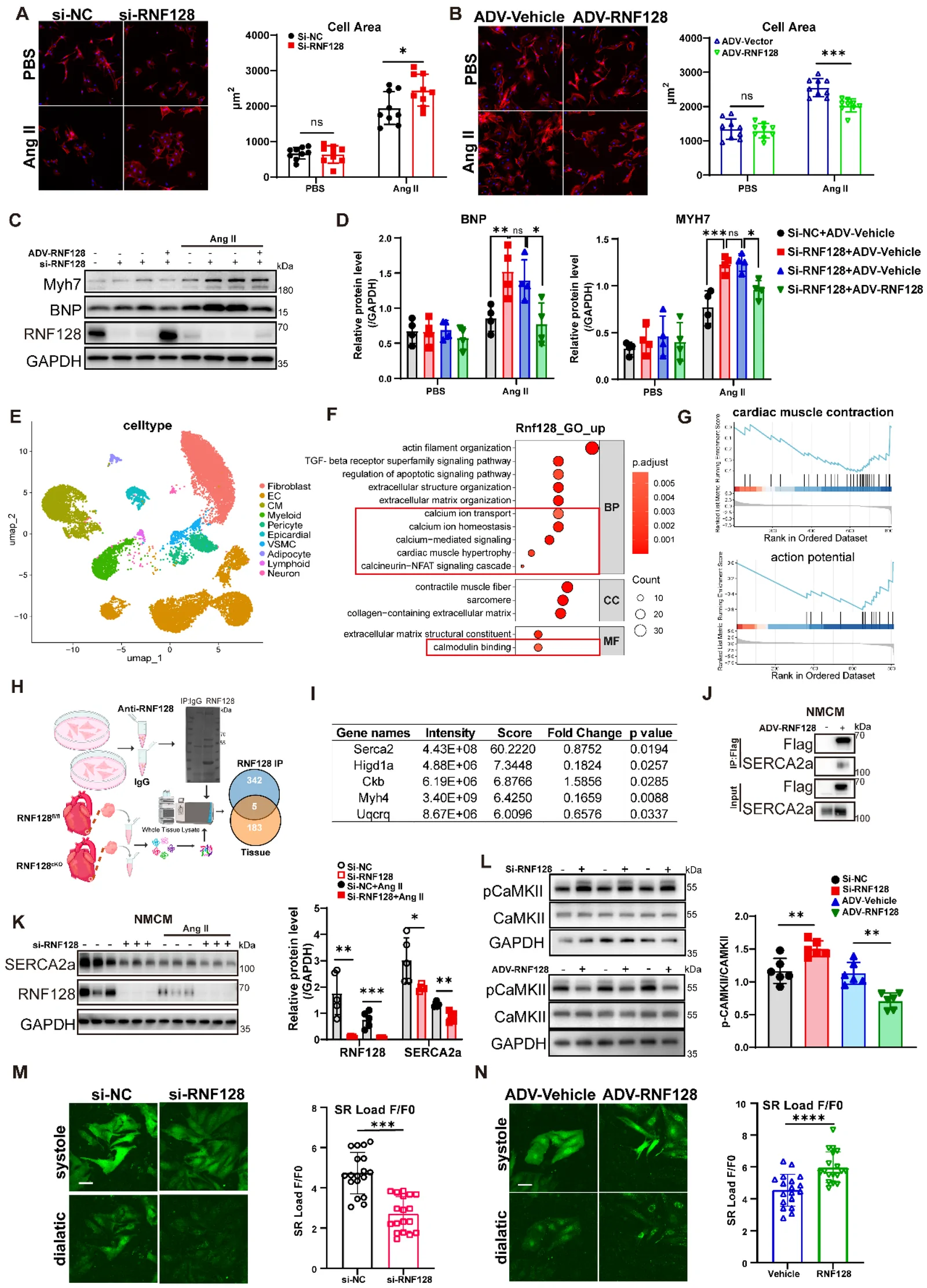
** RNF128 ameliorates cardiomyocyte hypertrophy and maintains calcium metabolism in cardiomyocytes. A** to **B**, neonatal mouse cardiomyocytes (NMCM) treated with PBS or Ang II stained by TRITC Phalloidin and cell area quantification (scale bar = 50 μm, n = 9 cells). **C** to **D**, Representative images of NMCM immunoblotting and relative protein expression of BNP and MYH7. **E**, the UMAP of 10 cell subtypes among TAC treated RNF128^fl/fl^ and RNF128^cKO^ mice. **F**, Gene Ontology (GO) pathway of upregulated genes in RNF128^cKO^ cardiomyocytes. **G**, Gene Set Enrichment Analysis (GSEA) of cardiac muscle contraction and action potential pathway. **H**, Workflow diagram of protein profiling and multi-omics sequencing. **I**, five common genes of altered proteins and RNF128 interacting proteins (Gene names: names of common proteins; Intensity: intensity of interacting protein mass spectrometry; Score: score of Co-IP mass spectrometry; Fold Change: expression fold change in protein profiling of heart tissue; p-value: heart tissue protein profiling).** J**, Coimmunoprecipitation of RNF128 and SERCA2a in NMCMs.** K**, Representative immunoblotting image of SERCA2a and RNF128 in NMCMs (n = 5 biological replicates, one-way ANOVA). **L**, Representative western blotting results of phospho-CaMKII and total CaMKII (Calcium/calmodulin-dependent Protein Kinase II). The protein level was standardized by CaMKII (n = 6 biological replicates, Student’s t-test, Si-NC vs Si-RNF128, ADV-Vehicle vs ADV-RNF128). **M** to **N**, Cytosolic Ca^2+^ determined by 2μM Fluo-4 AM fluorescence in NMCMs and quantification of SR load (n = 18 cells, Student’s t-test). (ns p > 0.05, * p < 0.05, ** p < 0.01, *** p < 0.001, **** p < 0.0001.)

Phalloidin staining and cell-area quantification of isolated NMCMs suggested an anti-hypertrophic effect of RNF128 *in vitro* (Figure 4A-B). Silencing RNF128 by small interfering RNA (siRNA) upregulated expression of the cardiac hypertrophic biomarkers ANP, BNP and MYH7. Overexpressing RNF128 could reverse this trend (Figure S4A, Figure 4C-D).

To explore the underlying molecular mechanism of RNF128 in cardiomyocytes, scRNA-seq was conducted using hearts from RNF128^cKO^ and RNF128^fl/fl^ mice. Following quality control and data preprocessing, we captured and mixed a total of 10,000 nuclei in one sample from 3 mice per group and identified 10 distinct cell clusters (Figure 4E). We then performed GO (Gene Ontology) enrichment analysis of upregulated genes in cardiomyocytes from RNF128^cKO^ samples, and found that intracellular calcium-related signaling pathways were enriched in RNF128^cKO^ cardiomyocytes (Figure 4F). GSEA (Gene Set Enrichment Analysis) further indicated signaling pathways directly associated with intracellular calcium homeostasis, including cardiac muscle contraction and action potential, were significantly downregulated (Figure 4G). In contrast, signaling pathways involved in Ras, apoptosis, MAPK and PI3K were markedly upregulated, while mitochondrial function and oxidative phosphorylation were impaired in RNF128^cKO^ cardiomyocytes (Figure S4B). Collectively, these findings at the single-cell level corroborate that RNF128 deficiency significantly disrupts the normal physiological function of cardiomyocytes.

To identify RNF128-associated downstream targets, Flag-labeled RNF128 was transfected into NMCMs by adenovirus (ADV). Cell lysate was subjected to Co-IP (coimmunoprecipitation), coupled with LC-MS (liquid chromatography-mass spectrometry). The IgG group served as the negative control. After excluding proteins detected in the IgG group, 347 RNF128 interacting proteins were detected. We also collected tissues from RNF128^fl/fl^ and RNF128^cKO^ mice after TAC surgery and identified 188 proteins with altered expression in tissue samples (Figure 4H). Five overlapping proteins were listed as candidate targets for RNF128. The five identified proteins were ranked according to mass spectrometric score, among which SERCA2 had the highest score (Figure 4I, Figure S4C). In line with the single-cell findings, SERCA2 is strongly associated with cardiomyocyte calcium transients 24, fibrotic activation 25, and signaling pathway activation 26. Accordingly, SERCA2 was chosen as the prime target for follow-up verification experiments. The protein-protein interaction between RNF128 and SERCA2 was confirmed in NMCMs (Figure 4J). As SERCA2a accounts for the dominant isoform expressed in cardiomyocytes, we constructed Myc-tagged SERCA2a and Flag-tagged RNF128 plasmids and performed reciprocal co-immunoprecipitation to validate their interaction in HEK293T (Figure S4D). In NMCMs, silencing RNF128 significantly downregulated SERCA2a at the protein level but not at the mRNA level (Figure 4K, Figure S4E), indicating a post-transcriptional regulatory pattern.

The phosphorylation level of CaMKII serves as a critical indicator of cytosolic calcium overload in cardiomyocytes. Knockdown of RNF128 significantly increased the p-CaMKII/total CaMKII ratio, whereas RNF128 overexpression exerted the opposite effect (Figure 4L). We used the Fluo-4 AM fluorescent probe to directly detect intracellular calcium transients. In NMCMs, F/F₀ ratio indicated altered sarcoplasmic reticulum (SR) Ca²⁺ load after silencing or overexpressing RNF128 (Figure 4M-N). We also measured intracellular calcium transient in primary adult mouse cardiomyocytes (AMCM) using the IonOptix system. After 4 weeks of Ang II administration, cardiomyocytes isolated from RNF128^cKO^ mice exhibited a longer decay constant and lower peak Ca^2+^ transient amplitude (peak h) than RNF128^fl/fl^ mice (Figure S4F-H), together with lower calcium return velocity (Figure S4I). The results suggested RNF128 might modulate impaired sarcoplasmic calcium restoration and prolonged relaxation phase through SERCA2a.

### RNF128 directly catalyzes SERCA2a’s K63-linked ubiquitination and prevents its degradation

**Figure 5:**
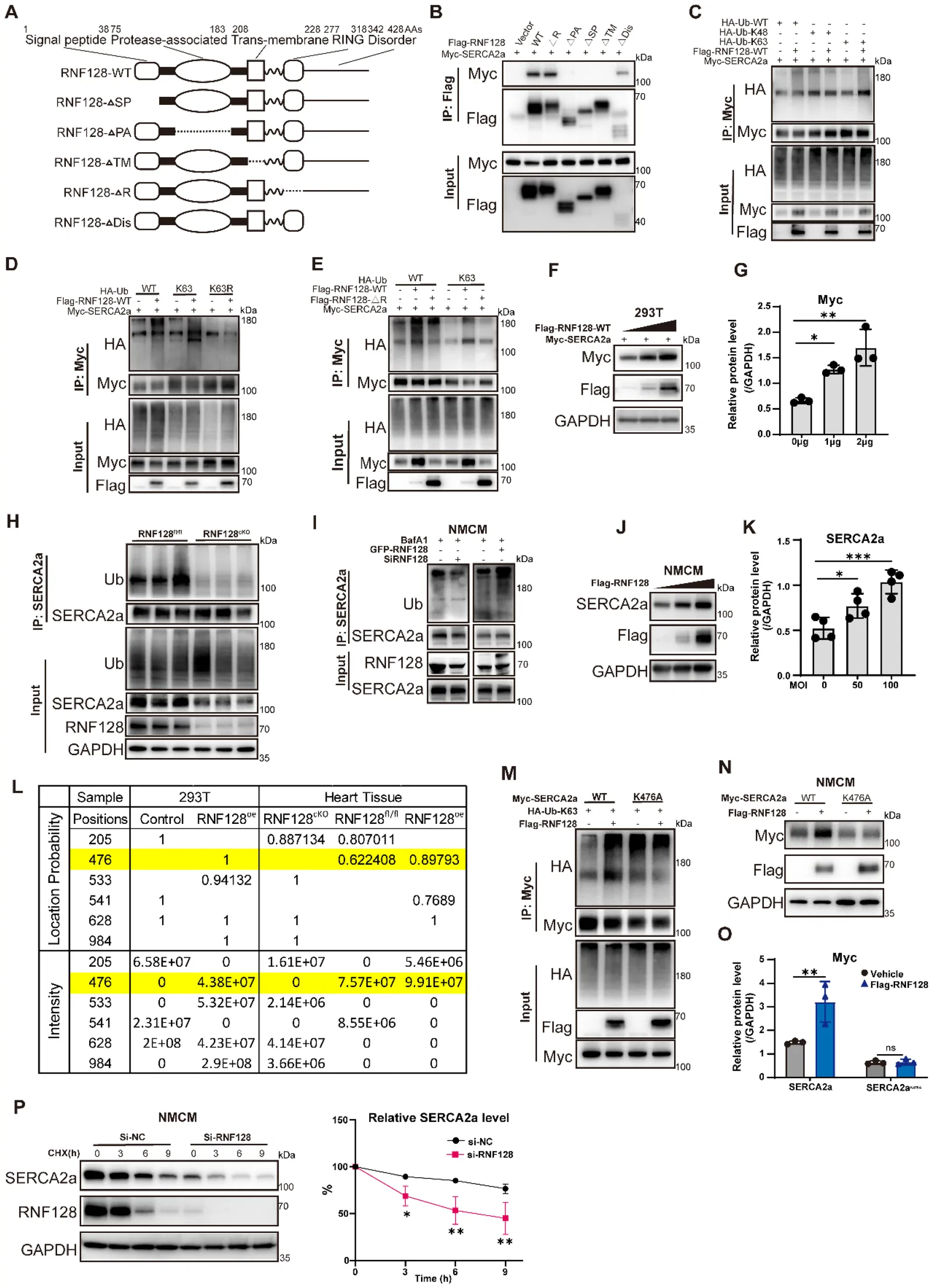
** RNF128 directly catalyzes SERCA2a’s K63-linked ubiquitination and prevents its degradation. A**, Schematic illustration of 6 mutants of RNF128 plasmids. **B**, Coimmunoprecipitation of Flag-tagged RNF128 mutants and Myc-tagged SERCA2a in HEK293T (293T) cells. **C**, RNF128 catalyzes K63, not K48 polyubiquitination of SERCA2a. **D**, the regulatory effect of RNF128 on SERCA2a ubiquitination is completely abolished when ubiquitin is mutated at lysine 63 (K63R). **E**, the catalytic function of RNF128 requires RING domain.** F** to **G**, RNF128 increased SERCA2a’s stability in a dose-dependent way in 293T cells (n = 3 biological replicates, vs 0 μg, one-way ANOVA). **H**, RNF128 catalyzed SERCA2a’s ubiquitination *in vivo*. **I**, RNF128 catalyzed SERCA2a’s ubiquitination in NMCMs.** J** to** K**, RNF128 increased SERCA2a’s stability in a concentration-dependent way in NMCMs cells (n = 4 biological replicates, vs 0 MOI, one-way-ANOVA).** L**, Common modification sites of SERCA2a in 293T cells and heart tissues (sites with localization probability > 0.75 were defined as confident identifications. Intensity indicates peak intensity of the peptide fragment containing the modification site). **M**, Lysine 476 to alanine mutation (K476A) abrogates RNF128-mediated ubiquitination of SERCA2a. **N** to **O,** K476A mutation eliminates RNF128’s protective effect on SERCA2a (n = 3 biological replicates, Student’s t-test, **O**).** P**, Representative western blot bands of SERCA2a from NMCMs undergoing 50 μM cycloheximide pulse-chase stability detection (n = 3 biological replicates, Two-way ANOVA followed by Šidák’s multiple-comparisons test to compare protein abundance across CHX time points between Si-NC and Si-RNF128 groups.). (ns, p > 0.05, * p < 0.05, ** p < 0.01, *** p < 0.001, **** p < 0.0001.)

To validate the hypothesis, we first determined the domains of RNF128 that were responsible for interaction with substrates and constructed different plasmids of RNF128 lacking each of the five domains (Figure 5A). The protein association (PA) domain and RING domain exhibit high evolutionary conservation across species. Previous studies established that RNF128 captures substrate proteins through its PA domain and catalyzes ubiquitin (Ub) conjugation via the RING domain 21. Co-transfection with Myc-SERCA2a into 293T cells followed by Flag-affinity immunoprecipitation revealed that RNF128-SERCA2a interaction requires intact PA domain and membrane localization (Figure 5B).

Subsequent Co-IP experiments in 293T cells co-expressing Myc-tagged SERCA2a, Flag-tagged RNF128, and HA-tagged ubiquitin (Ub) variants demonstrated that RNF128 enhanced SERCA2a’s ubiquitination (Figure 5C), particularly promoting K63-linked polyubiquitination (Figure 5D-E). RNF128’s stabilizing effects showed a dose-dependent effect in 293T (Figure 5F-G). RNF128 also catalyzed the ubiquitination of SERCA2a *in vivo* and NMCMs (Figure 5H-I), with similar stabilizing pattern in NMCMs (Figure 5J-K). Six overlapping ubiquitination sites on SERCA2a were identified via quantitative ubiquitinomic analysis in 293T cells and mouse cardiac tissues. The localization probability and signal intensity of the residue K476 was found to be positively associated with RNF128 expression (Figure 5L). Upon introduction of the K476A mutation, RNF128-mediated K63-linked ubiquitination of SERCA2a was eliminated (Figure 5M), and the protective effect of RNF128 on SERCA2a protein stability was abrogated (Figure 5N-O). We conducted cycloheximide (CHX, 50 μM) chase assay for 0, 3, 6 and 9 h and demonstrated that RNF128 significantly ameliorated the degradation of SERCA2a in NMCMs and 293T (Figure 5P, Figure S5A-B).

### RNF128 maintains calcium homeostasis in cardiomyocytes by suppressing autophagy-lysosome-mediated degradation of SERCA2a

**Figure 6:**
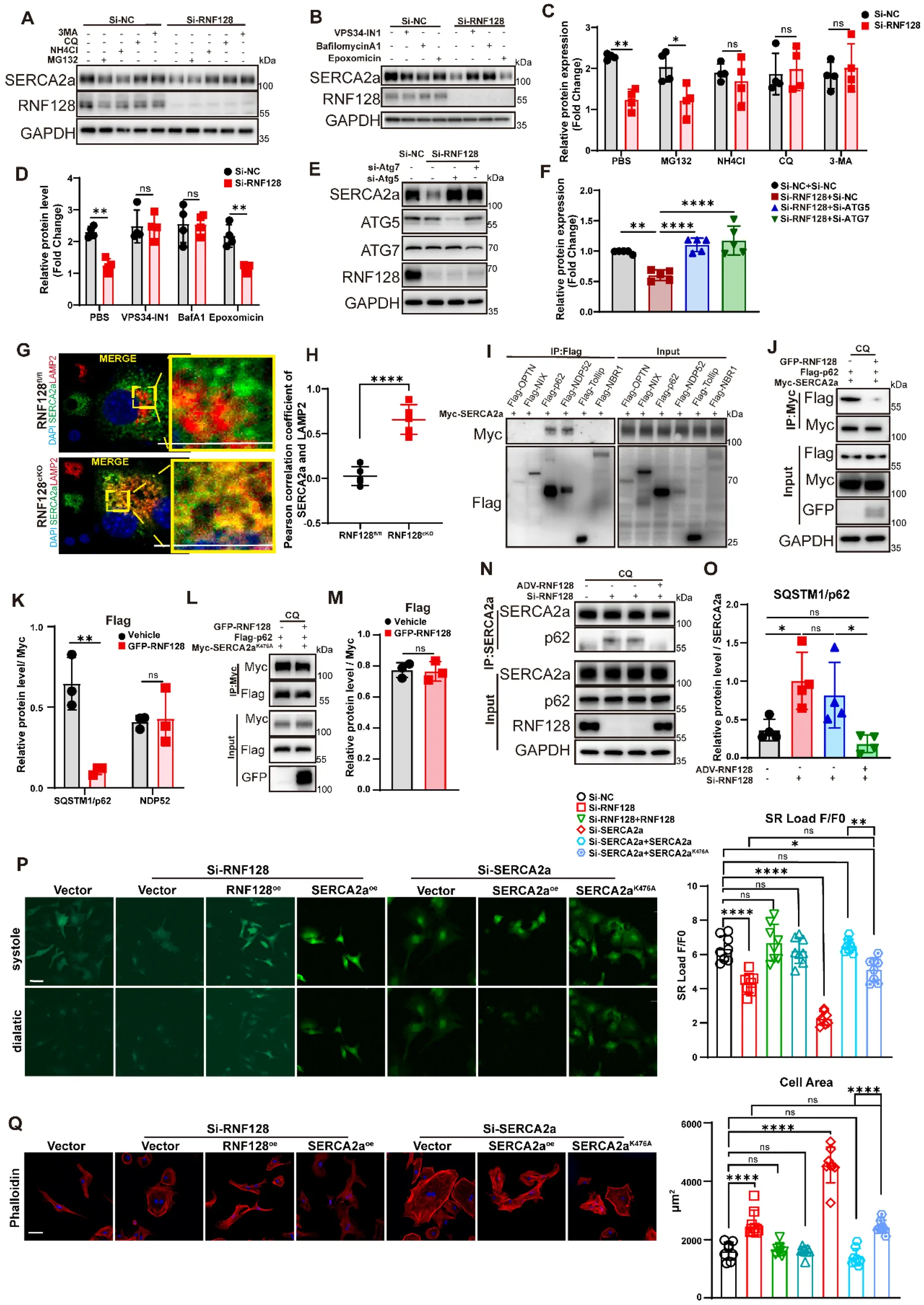
** RNF128 maintains calcium homeostasis in cardiomyocytes by suppressing autophagy-lysosome degradation of SERCA2a. A**, Representative western blotting for SERCA2a in NMCMs treated with MG132 (Carbobenzoxy-L-leucyl-L-leucyl-L-leucinal), CQ (Chloroquine), NH4Cl (Ammonium chloride), and 3-MA (3-Methyladenine) for 6 hours. **B**, Representative western blotting for SERCA2a in NMCMs treated with BafA1 (bafilomycin A1), VPS34-IN1 and Epoxomicin for 6 hours. **C** to **D**, Quantification of SERCA2a (n = 4 biological replicates, vs Si-NC of each inhibitor, two-way ANOVA). **E** to **F**, Silencing ATG5 or ATG7 inhibits SERCA2a’s degradation in RNF128 knockdown NMCMs (**F**, n = 5 biological replicates, vs Si-RNF128+Si-NC, one-way ANOVA). **G**, Immunofluorescence staining of SERCA2a and lysosome-associated membrane protein 2 (LAMP2, Scale bar = 2μm). **H**, Pearson correlation coefficient of SERCA2a and LAMP2 in RNF128^fl/fl^ and RNF128^cKO^ cardiomyocytes (n = 5 biological replicates, Student’s t-test). **I**, Representative western blotting of coimmunoprecipitation of myc-SERCA2a with Flag-tagged autophagy cargo receptors in 293T cells.** J**, Representative western blotting of coimmunoprecipitation of SERCA2a with Flag-tagged SQSTM1/p62 in 293T. **K**, RNF128 inhibits SQSTM1/p62-SERCA2a binding but not NDP52-SERCA2a interaction (n = 3 biological replicates, vs Vehicle of each group, Student’s t-test). **L** to **M**, K476A mutation abrogates the regulatory effect of RNF128 on p62-SERCA2a interaction (**M**, n = 3 biological replicates, Student’s t-test).** N** to **O**, Representative image of immunoblotting of coimmunoprecipitation of SERCA2a with SQSTM1/p62 in NMCMs (n = 4 biological replicates, one-way ANOVA). **P**, Fluo-4 AM fluorescence signal reflected cytosolic Ca^2+^ in NMCMs after treatment with siRNA and overexpressing plasmids (n = 8 cells, one-way ANOVA). **Q**, TRITC Phalloidin staining and corresponding quantification (scale bar = 50 μm, n = 8 cells from 4 biological replicates, one-way ANOVA). (ns p > 0.05, * p < 0.05, ** p < 0.01, *** p < 0.001, **** p < 0.0001.)

Prior research established that SERCA2a undergoes degradation predominantly via the ubiquitin-proteasome pathway, but some reports suggest ancillary lysosomal involvement 5, 8, 27. We treated NMCMs with proteasome inhibitors MG132 (Carbobenzoxy-L-leucyl-L-leucyl-L-leucinal, 50 μM, 6 h) and Epoxomicin versus lysosomal inhibitors Chloroquine (CQ, 50 μM, 6 h), Ammonium chloride (NH4Cl, 10 mM, 6 h), Bafilomycin (50 nM, 6 h) and autophagy inhibitor 3-Methyladenine (3-MA, 10 mM, 6 h), VPS34-IN1 (0.5 μM, 6 h) 28 (Figure S5C-D). Interestingly, immunoblotting showed that autophagy and lysosomal inhibitors, but not proteasome inhibitors, rescued SERCA2a degradation in RNF128 knockdown NMCMs (Figure 6A-D). Silencing ATG5 or ATG7 also inhibited SERCA2a degradation in RNF128-deficient cardiomyocytes (Figure 6E-F). SERCA2a and LAMP2 colocalization was significantly increased in RNF128^cKO^ NMCMs (Figure 6G-H). These results suggested that RNF128 might stabilize SERCA2a by suppressing its lysosomal degradation. Furthermore, we constructed six autophagy cargo-receptor plasmids and co-transfected them with myc-SERCA2a plasmid into 293T cells and discovered the interaction of NDP52 and SQSTM1/p62 with SERCA2a (Figure 6I). Further validation experiments demonstrated that p62-mediated recognition of SERCA2a was significantly attenuated upon RNF128 overexpression, while no significant alteration was detected for NDP52-mediated recognition (Figure 6J-K, Figure S5E, Figure 6N-O). The regulatory effect of RNF128 on p62-dependent SERCA2a recognition was abolished when the SERCA2a K476A mutation was introduced (Figure 6L–M). For functional rescue assays in RNF128-deficient NMCMs, ectopic expression of wild-type SERCA2a or RNF128 fully reversed the aberrant calcium transients and hypertrophic phenotype, whereas equivalent re-expression of the SERCA2a K476A mutant achieved partial phenotypic rescue (Figure 6P-Q). Collectively, these findings suggested that RNF128 regulates cardiomyocyte hypertrophy via the stabilization of SERCA2a at the cellular level.

### RNF128 rescues cardiac dysfunction and pathological remodeling through SERCA2a *in vivo*

**Figure 7:**
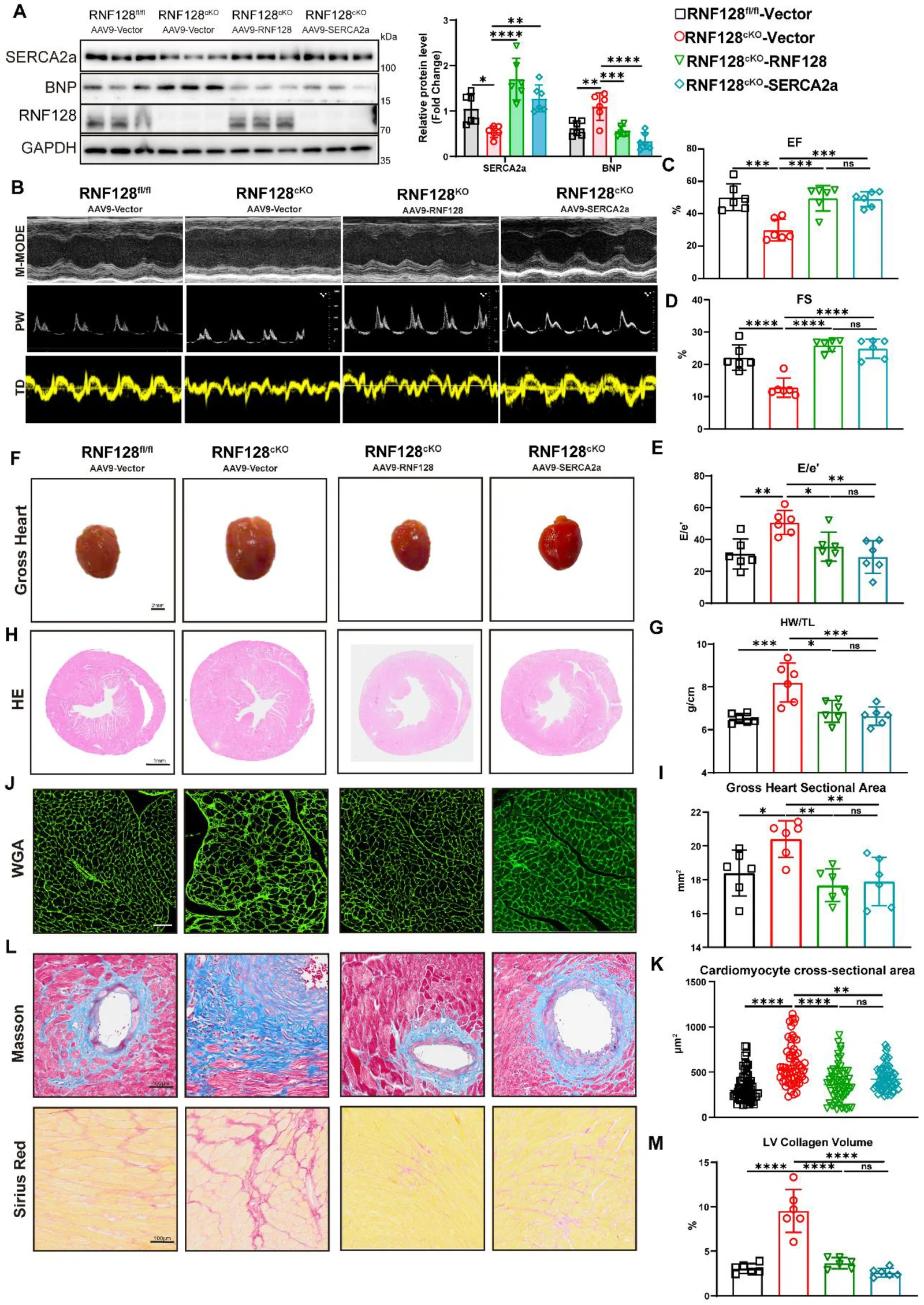
** RNF128 rescues cardiac dysfunction and pathological remodeling through SERCA2a *in vivo*. A**, Representative western blotting of cardiac tissues from RNF128^fl/fl^ or RNF128^cKO^ mice injected with AAV9-Vector/ AAV9-RNF128/ AAV9-SERCA2a. **B** to** E**, cardiac dysfunction was attenuated by RNF128 or SERCA2a restoration. M-mode images of each group (**B**), EF (**C**), FS (**D**) and E/e’ (**E**) quantification. **F**, Whole heart images (scale bar = 2 mm). **G,** Cardiac hypertrophy indicated by HW/TL was reduced after restoration of RNF128 or SERCA2a. **H**, Representative Hematoxylin–eosin (H&E) images. **I**, Quantification of heart sectional area. **J** to **K**, WGA staining and quantification of cardiomyocyte cross-sectional area (**K,** scale bar = 50 μm, n = 60 cells from 6 mice per group, Kruskal–Wallis H test with Mann–Whitney U test post-hoc pairwise comparisons). **L** to** M**, Masson and Sirius Red staining of heart sections and quantification of left ventricular fibrosis. (**A**, **C**, **D**, **E**, **G**, **I**, **M**, Bar graphs represent mean ± SD, n = 6 mice per group, vs RNF128^cKO^ + AAV9-Vector, one-way ANOVA with Dunnett’s post-hoc test was used for intergroup comparisons, with adjusted p-values displayed for multiple testing. ns, p > 0.05, * p < 0.05, ** p < 0.01, *** p < 0.001, **** p < 0.0001.)

In RNF128^cKO^ mice, cardiac-specific overexpression of RNF128 or SERCA2a via cTnT promoter-driven adeno-associated virus serotype 9 (AAV9) was performed (Figure S6A-B) and significantly restored SERCA2a protein levels (Figure 7A). AAV9 injection did not affect murine blood pressure (Figure S6C). After Ang II-induced modeling, both RNF128 and SERCA2a restoration significantly ameliorated cardiac dysfunction. Echocardiographic analysis demonstrated marked improvements in EF and FS (Figure 7B-E), accompanied by reduced LVPW thickness in rescue groups (Table S3). Moreover, RNF128 or SERCA2a restoration attenuated pathological cardiac remodeling, as evidenced by decreased HW/TL and HW/BW and reduced cardiac dimensions (Figure 7F-G, Figure S6D). Cardiomyocyte cross-sectional areas were diminished in rescue groups (Figure 7H-K). Consistently, Masson and Sirius Red staining confirmed reduced LV fibrosis (Figure 7L-M). qRT-PCR further validated downregulation of ventricular remodeling markers in treated cohorts (Figure S6E). Serum ELISA showed lower ANP and BNP levels after restoration of RNF128 or SERCA2a in RNF128^cKO^ mice (Figure S6F). IHC staining showed lower BNP and MYH7 content in RNF128 or SERCA2a restored mice (Figure S6G).

### RNF128 overexpression mitigates Ang II-induced cardiac hypertrophy and cardiac dysfunction

**Figure 8:**
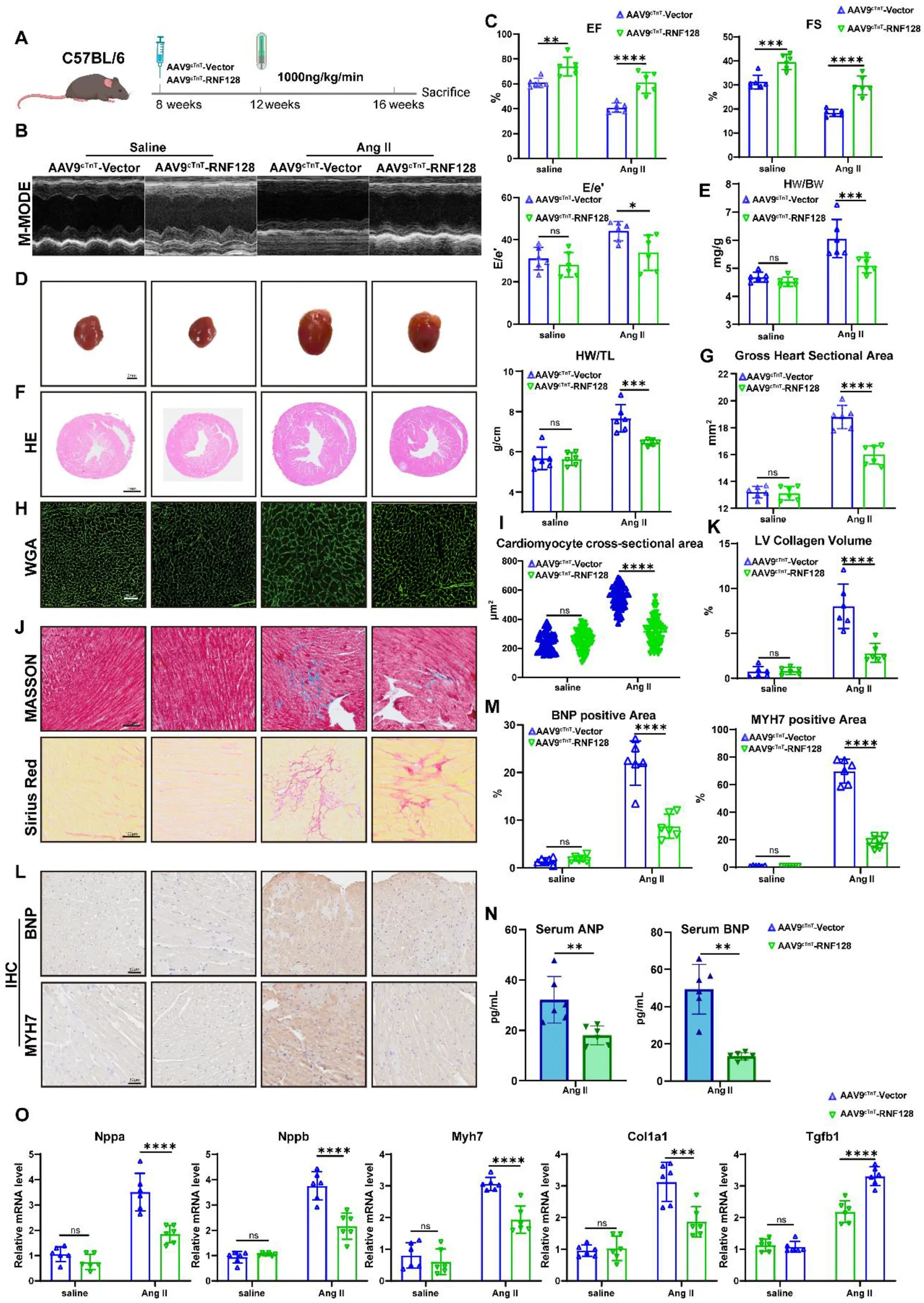
** RNF128 overexpression relieves Ang II-induced hypertrophic remodeling. A**, Flowchart for cTnT promote-driven Adeno-Associated Virus Serotype 9 (AAV9^cTnT^) injection followed by Ang II-infusion. **B,** Representative M-mode images. **C**, EF, FS and E/e’. **D**, RNF128 upregulation mitigates Ang II-induced cardiac enlargement (scale bar = 2 mm). **E**, RNF128 upregulation reverses the increased HW/BW and HW/TL ratios triggered by cardiac hypertrophy. **F** to **G**, H&E staining showing gross heart sectional area. **H** and** I**, WGA staining showed decreased cardiomyocyte cross-sectional area after RNF128 overexpression (n = 60 cells from 6 mice per group, Kruskal–Wallis H test, and post-hoc pairwise comparisons were conducted with the Mann–Whitney U test). **J** to** K**, Masson and Sirius Red in sections of hearts suggesting reduced left ventricular fibrosis in RNF128^oe^ mice. **L** to** M**, Immunohistochemical (IHC) of BNP and MYH7 in heart sections. **N**, The serum ANP and BNP concentration. (n = 6 mice for each group, Welch’s t-test). **O**, Real-time qPCR analysis of *Nppa*, *Nppb*, *Myh7*, *Col1a1* and *Tgfb1* in heart tissues. **(C**,** E**,** G**,** K**,** M**,** O**, Bar graphs represent mean ± SD, n = 6 mice per group, Šídák’s multiple comparisons test was performed after two-way ANOVA for VECTOR and RNF128 mice within saline or Ang II group. Adjusted p-values were displayed for multiple testing. mRNA abundance in each experimental group was normalized to the AAV9-VECTOR + saline, defined as 1. ns p > 0.05, * p < 0.05, ** p < 0.01, *** p < 0.001, **** p < 0.0001.)

As a potent gene delivery system, AAV is increasingly used in gene therapy for various human diseases 29. Intravenous injection of cTnT-AAV9 was applied to induce cardiomyocyte-specific RNF128 overexpression in *C57BL/6* mice. Ang II infusion was initiated 4 weeks after AAV9 administration (Figure 8A). Blood pressure was monitored weekly (Figure S7A). Transduction efficiency was validated at the mRNA and histological levels (Figure S7B-D). Ang II treatment triggered a prominent decline in EF among AAV9-Vector mice. In contrast, AAV9-RNF128 mice had higher EF values after Ang II treatment. Parallel changes were observed in the E/e’ ratio, indicating that RNF128 overexpression preserved cardiac function under Ang II-induced stress (Figure 8B-C, Table S4).

Concurrently, AAV9-RNF128 mice exhibited markedly attenuated cardiac hypertrophy (Figure 8D-I), along with significantly reduced myocardial interstitial fibrosis (Figure 8J-K). Immunohistochemical staining of cardiac sections from RNF128-overexpressing mice showed reduced levels of BNP and MYH7 (Figure 8L-M), and serum ANP and BNP were lower in the RNF128-overexpressing mice (Figure 8N). RNF128 overexpression also significantly suppressed the expression of hypertrophy-associated genes (Figure 8O).

Collectively, our data demonstrate that RNF128 ameliorates pathological cardiac hypertrophy by maintaining intracellular calcium homeostasis. Mechanistically, the interaction between RNF128 and SERCA2a facilitates SERCA2a stabilization via K63-linked polyubiquitination. RNF128 disrupts SERCA2a’s association with autophagy cargo receptor p62 and inhibits its degradation via the autophagy-lysosome pathway. Critically, cardiomyocyte-specific overexpression of RNF128 *in vivo* conferred a sufficient therapeutic benefit against the progression of cardiac hypertrophy, highlighting its value as an innovative therapeutic target.

## Discussion

Pathological cardiac hypertrophy constitutes a critical pathological component in heart failure pathogenesis 2. Recent research has focused on elucidating pivotal molecular targets and delineating associated signal transduction mechanisms 30. Accumulating studies on pathological cardiac remodeling and dysfunction have revealed aberrant ubiquitination of a variety of key proteins, and multiple members of the RNF E3 ubiquitin ligase family — including RNF207 23 and RNF5 31 — exert pivotal regulatory roles in these pathological processes.

In this work, we combined public dataset mining and scRNA-seq analysis to pinpoint cardiomyocyte-specific RNF128 — a molecule with significantly dysregulated expression during pathological cardiac hypertrophy progression — as a novel therapeutic target. The protective function of RNF128 against cardiac hypertrophy and cardiac dysfunction was verified in cardiomyocyte-specific RNF128^cKO^ mouse models. Co-IP combined with LC-MS/MS and proteomic screening identified SERCA2a as the downstream effector of RNF128, with the underlying mechanism corroborated by systematic site-directed mutagenesis and *in vitro*/*in vivo* rescue assays. Furthermore, cardiomyocyte-specific overexpression of RNF128 mediated by cTnT promoter-driven AAV9 achieved pronounced therapeutic efficacy in ameliorating pathological cardiac remodeling.

RNF128 was initially identified as a specific mediator of CD4+ T lymphocyte anergy 32. To date, RNF128 has been studied and reported in various disease models, including cancer and inflammatory disease 22, 33, 34. In this work, cardiomyocyte-specific RNF128 ablation triggered enhanced inflammatory activation in cardiac tissue, manifesting as increased immune cell infiltration and an expanded pro-inflammatory monocyte fraction. This suggests that in addition to its direct functions in immune cells, RNF128 shapes the cardiac immune microenvironment through cardiomyocyte-immune cell crosstalk (Figure S8A-D).

We identified SERCA2a as a binding partner of RNF128. SERCA2a is the major isoform expressed in cardiomyocytes and mediates calcium reuptake 3. Its dysregulated expression or dysfunction directly causes aberrant calcium transients in cardiomyocytes 13, 26, 35. Excessive cytosolic calcium accumulation directly activates an array of downstream signaling pathways 30, including hypertrophic, profibrotic, and pro-inflammatory signaling cascades 36. This dysregulated calcium-dependent signaling activation acts as a key driver underlying the pathogenesis and progressive worsening of myocardial pathologies 5, 37.

Among all identified degradative pathways for SERCA2a, K48-linked polyubiquitination followed by proteasome-mediated breakdown has received the broadest experimental validation 5,6,38. Notably, our study demonstrates that RNF128 mediates K63-linked polyubiquitination of SERCA2a specifically at residue K476, and enhances its protein stability. Further functional validation confirms that RNF128-mediated K63-linked polyubiquitination weakened the interaction between SERCA2a and the cargo receptor SQSTM1/p62 and protected SERCA2a from lysosomal and autophagic degradation, consistent with the observation that SERCA2a turnover upon RNF128 ablation is largely blocked by autophagy-lysosome pathway inhibition. Unlike K48-linked ubiquitination, which primarily directs target proteins for proteasomal degradation, K63-linked ubiquitination exerts far more complex and diverse regulatory effects on protein function and stability, such as mediating protein translocation 16, protein stability 17 or protein complex formation 18. Moreover, K63-linked polyubiquitination can function as a selective degradation signal recognized by the autophagy cargo p62 to facilitate substrate clearance 38-42. Conversely, it may also serve as a protective modification that triggers conformational alterations of the target protein or masks its intrinsic degradation recognition interface, thereby inhibiting protein breakdown 21, 43, 44. While the exact structural basis for this protective effect remains to be determined, we speculate that K63-linked ubiquitination at K476 may induce conformational changes in SERCA2a that mask its p62-recognition interface. Future structural studies will be required to directly test this hypothesis.

One limitation of the present study is the control-group design of the mouse model. RNF128 ^cKO^ mice were generated by crossing Myh6-Cre and RNF128^fl/fl^ on a *C57BL/6J* background. While prior studies have reported age-dependent intrinsic cardiotoxicity of Myh6-Cre 45, using RNF128^fl/fl^ mice as the control for the RNF128^cKO^ mice may not fully rule out the non-specific phenotypic effects induced by Cre recombinase. Nevertheless, all experiments were terminated at 3 months of age, prior to the typical onset of Cre-related cardiac impairment, consistent with the widespread use of this Cre line in young adult mouse studies 14, 40. Furthermore, cardiomyocyte-specific overexpression of RNF128 via cTnT promoter-driven AAV9 in *C57BL/6J* mice with no Myh6-Cre background exerted pronounced cardioprotective effects. To further exclude potential Cre-mediated confounding effects on our phenotypes, we included a control cohort of 2-month-old Myh6-Cre mice. Four weeks after TAC surgery, no statistically significant differences were observed in cardiac function, HW/BW or HW/TL between Myh6-Cre mice and RNF128^fl/fl^. Both control groups showed significant differences in these parameters compared with RNF128^cKO^ mice (Figure S8E). These findings confirmed that the observed cardiac phenotypes were predominantly driven by the loss of RNF128 in cardiomyocytes, rather than the intrinsic cardiotoxicity of Myh6-Cre itself.

Another limitation of this study is the lack of identification of deubiquitinases (DUBs) in the site-specific ubiquitination regulation of SERCA2a. Protein ubiquitination status is governed by the dynamic balance between ubiquitin ligases and deubiquitinases; our work has characterized RNF128 as the E3 ligase mediating K63-linked polyubiquitination of SERCA2a at residue K476, but the DUB that reverses this specific modification remains unidentified. Although previous studies have reported several DUBs that modulate the overall ubiquitination level and stability of SERCA2a 5, 14, 15, it remains unclear whether these enzymes target the Lys476 residue and specifically edit K63-linked ubiquitin chains. The absence of this information limits the complete delineation of the regulatory signaling cascade upstream of SERCA2a degradation and hinders a full understanding of the dynamic control of calcium handling in cardiomyocytes. We will prioritize the screening and functional validation of site-specific DUBs targeting SERCA2a Lys476 in follow-up studies to refine the complete regulatory network.

In summary, RNF128 was identified as a suppressor of pathological cardiac hypertrophy. RNF128 inhibits lysosomal-mediated degradation of SERCA2a by catalyzing its K63-linked ubiquitination, thereby affecting intracellular calcium transients and calcium homeostasis. These findings highlight the translational potential of RNF128 as a novel therapeutic target to attenuate hypertrophic progression and preserve cardiac function.

## Supplementary Material

Supplementary Figures S1-S8. Tables S1-S4 present cardiac function, Table S5 presents peptides of SERCA2a detected by mass spectrometry. Table S6 presents clinical information of human samples. Description of the remaining materials and methods.

## Data Availability

All data supporting the paper’s conclusions are included in the main manuscript or available upon reasonable request from corresponding author.

## Abbreviations

RNF128: ring finger protein 128
SERCA2a: sarcoplasmic/endoplasmic reticulum calcium ATPase 2a
TAC: transverse aortic constriction
Ang II: angiotensin II
WGA: wheat germ agglutinin
HW/BW: heart weight / body weight
HW/TL: heart weight / tibial length
ANP: atrial natriuretic peptide
BNP: brain natriuretic peptide
MYH7: myosin heavy chain 7
CaMKII: calcium/calmodulin-dependent protein kinase II
NMCM: neonatal mouse cardiomyocyte
EF: ejection fraction
FS: fractional shortening
E/e’: early diastolic mitral inflow velocity to early diastolic mitral annular velocity ratio
AAV9: adeno-associated virus serotype 9

## References

[1] Guo J, Mihic A, Wu J, Zhang Y, Singh K, Dhingra S (2015). Canopy 2 attenuates the transition from compensatory hypertrophy to dilated heart failure in hypertrophic cardiomyopathy. Eur Heart J.

[2] Nakamura M, Sadoshima J (2018). Mechanisms of physiological and pathological cardiac hypertrophy. Nat Rev Cardiol.

[3] Vangheluwe P, Raeymaekers L, Dode L, Wuytack F (2005). Modulating sarco(endo)plasmic reticulum Ca2+ ATPase 2 (SERCA2) activity: cell biological implications. Cell Calcium.

[4] Torre E, Arici M, Lodrini AM, Ferrandi M, Barassi P, Hsu SC (2022). SERCA2a stimulation by istaroxime improves intracellular Ca2+ handling and diastolic dysfunction in a model of diabetic cardiomyopathy. Cardiovasc Res.

[5] Ye B, Zhou H, Chen Y, Luo W, Lin W, Zhao Y (2023). USP25 Ameliorates Pathological Cardiac Hypertrophy by Stabilizing SERCA2a in Cardiomyocytes. Circ Res.

[6] Yang N, Li L, Shi XL, Liu YP, Wen R, Yang YH (2025). Succinylation of SERCA2a at K352 Promotes Its Ubiquitinoylation and Degradation by Proteasomes in Sepsis-Induced Heart Dysfunction. Circ Heart Fail.

[7] Roh JD, Hobson R, Chaudhari V, Quintero P, Yeri A, Benson M (2019). Activin type II receptor signaling in cardiac aging and heart failure. Sci Transl Med.

[8] Lei D, Liu Y, Liu Y, Jiang Y, Lei Y, Zhao F (2025). The gut microbiota metabolite trimethylamine N-oxide promotes cardiac hypertrophy by activating the autophagic degradation of SERCA2a. Commun Biol.

[9] Gubas A, Dikic I (2022). ER remodeling via ER-phagy. Mol Cell.

[10] Zsebo K, Yaroshinsky A, Rudy JJ, Wagner K, Greenberg B, Jessup M (2014). Long-term effects of AAV1/SERCA2a gene transfer in patients with severe heart failure: analysis of recurrent cardiovascular events and mortality. Circ Res.

[11] Arici M, Hsu SC, Ferrandi M, Barassi P, Ronchi C, Torre E (2024). Selective SERCA2a activator as a candidate for chronic heart failure therapy. J Transl Med.

[12] Gorski PA, Jang SP, Jeong D, Lee A, Lee P, Oh JG (2019). Role of SIRT1 in Modulating Acetylation of the Sarco-Endoplasmic Reticulum Ca(2+)-ATPase in Heart Failure. Circ Res.

[13] Kho C, Lee A, Jeong D, Oh JG, Chaanine AH, Kizana E (2011). SUMO1-dependent modulation of SERCA2a in heart failure. Nature.

[14] Han J, Fang Z, Han B, Ye B, Lin W, Jiang Y (2023). Deubiquitinase JOSD2 improves calcium handling and attenuates cardiac hypertrophy and dysfunction by stabilizing SERCA2a in cardiomyocytes. Nat Cardiovasc Res.

[15] Huang Z, Han X, Hou Y, Wang X, Qiu F, Gong Y (2026). Cardiomyocyte-derived OTUD7B promotes cardiac hypertrophy by deubiquitinating SERCA2a. Theranostics.

[16] Yu J, Li J, Shen A, Liu Z, He TS (2024). E3 ubiquitin ligase RNF128 negatively regulates the IL-3/STAT5 signaling pathway by facilitating K27-linked polyubiquitination of IL-3Ralpha. Cell Commun Signal.

[17] Song G, Liu B, Li Z, Wu H, Wang P, Zhao K (2016). E3 ubiquitin ligase RNF128 promotes innate antiviral immunity through K63-linked ubiquitination of TBK1. Nat Immunol.

[18] Nurieva RI, Zheng S, Jin W, Chung Y, Zhang Y, Martinez GJ (2010). The E3 ubiquitin ligase GRAIL regulates T cell tolerance and regulatory T cell function by mediating T cell receptor-CD3 degradation. Immunity.

[19] Wei CY, Zhu MX, Yang YW, Zhang PF, Yang X, Peng R (2019). Downregulation of RNF128 activates Wnt/beta-catenin signaling to induce cellular EMT and stemness via CD44 and CTTN ubiquitination in melanoma. J Hematol Oncol.

[20] Liu PY, Chen CC, Chin CY, Liu TJ, Tsai WC, Chou JL (2021). E3 ubiquitin ligase Grail promotes hepatic steatosis through Sirt1 inhibition. Cell Death Dis.

[21] Liu Y, Zhang X, Yu L, Cao L, Zhang J, Li Q (2025). E3 ubiquitin ligase RNF128 promotes Lys63-linked polyubiquitination on SRB1 in macrophages and aggravates atherosclerosis. Nat Commun.

[22] He TS, Cai K, Lai W, Yu J, Qing F, Shen A (2024). E3 ubiquitin ligase RNF128 attenuates colitis and colorectal tumorigenesis by triggering the degradation of IL-6 receptors. J Adv Res.

[23] Yuan L, Bu S, Du M, Wang Y, Ju C, Huang D (2023). RNF207 exacerbates pathological cardiac hypertrophy via post-translational modification of TAB1. Cardiovasc Res.

[24] Cutler MJ, Wan X, Plummer BN, Liu H, Deschenes I, Laurita KR (2012). Targeted sarcoplasmic reticulum Ca2+ ATPase 2a gene delivery to restore electrical stability in the failing heart. Circulation.

[25] Chen W, Liu L, Tang M, Li J, Yuan W, Yin D (2024). Type I collagen-targeted liposome delivery of Serca2a modulates myocardium calcium homeostasis and reduces cardiac fibrosis induced by myocardial infarction. Mater Today Bio.

[26] Goodman JB, Qin F, Morgan RJ, Chambers JM, Croteau D, Siwik DA (2020). Redox-Resistant SERCA [Sarco(endo)plasmic Reticulum Calcium ATPase] Attenuates Oxidant-Stimulated Mitochondrial Calcium and Apoptosis in Cardiac Myocytes and Pressure Overload-Induced Myocardial Failure in Mice. Circulation.

[27] Santiago-Fernandez O, Coletto L, Tasset I, Kaushik S, Concepcion AR, Qaisar R (2025). Age-related decline of chaperone-mediated autophagy in skeletal muscle leads to progressive myopathy. Nat Metab.

[28] Wu X, Zheng Y, Liu M, Li Y, Ma S, Tang W (2021). BNIP3L/NIX degradation leads to mitophagy deficiency in ischemic brains. Autophagy.

[29] Wang JH, Gessler DJ, Zhan W, Gallagher TL, Gao G (2024). Adeno-associated virus as a delivery vector for gene therapy of human diseases. Signal Transduct Target Ther.

[30] Kreusser MM, Lehmann LH, Keranov S, Hoting MO, Oehl U, Kohlhaas M (2014). Cardiac CaM Kinase II genes delta and gamma contribute to adverse remodeling but redundantly inhibit calcineurin-induced myocardial hypertrophy. Circulation.

[31] Yang LL, Xiao WC, Li H, Hao ZY, Liu GZ, Zhang DH (2022). E3 ubiquitin ligase RNF5 attenuates pathological cardiac hypertrophy through STING. Cell Death Dis.

[32] Whiting CC, Su LL, Lin JT, Fathman CG (2011). GRAIL: a unique mediator of CD4 T-lymphocyte unresponsiveness. FEBS J.

[33] Liu PY, Chen CY, Lin YL, Lin CM, Tsai WC, Tsai YL (2023). RNF128 regulates neutrophil infiltration and myeloperoxidase functions to prevent acute lung injury. Cell Death Dis.

[34] Ran X, Li Y, Ren Y, Chang W, Deng R, Wang H (2025). RNF128 deficiency in macrophages promotes colonic inflammation by suppressing the autophagic degradation of S100A8. Cell Death Dis.

[35] Stege NM, de Boer RA, Makarewich CA, van der Meer P, Sillje HHW (2024). Reassessing the Mechanisms of PLN-R14del Cardiomyopathy: From Calcium Dysregulation to S/ER Malformation. JACC Basic Transl Sci.

[36] Kim OH, Noh SW, Choi JS, Jung Y, Oh BC (2025). The SERCA-PLN-DWORF axis in cardiometabolic disease: mechanisms and therapeutic perspectives. Cardiovasc Diabetol.

[37] Li X, La Salvia S, Liang Y, Adamiak M, Kohlbrenner E, Jeong D (2023). Extracellular Vesicle-Encapsulated Adeno-Associated Viruses for Therapeutic Gene Delivery to the Heart. Circulation.

[38] Yang C, Wang Z, Kang Y, Yi Q, Wang T, Bai Y (2023). Stress granule homeostasis is modulated by TRIM21-mediated ubiquitination of G3BP1 and autophagy-dependent elimination of stress granules. Autophagy.

[39] Wang B, Jie Z, Joo D, Ordureau A, Liu P, Gan W (2017). TRAF2 and OTUD7B govern a ubiquitin-dependent switch that regulates mTORC2 signalling. Nature.

[40] Xie W, Tian S, Yang J, Cai S, Jin S, Zhou T (2022). OTUD7B deubiquitinates SQSTM1/p62 and promotes IRF3 degradation to regulate antiviral immunity. Autophagy.

[41] Zhou X, Zhao Y, Huang S, Shu H, Zhang Y, Yang H (2025). TRIM32 promotes neuronal ferroptosis by enhancing K63-linked ubiquitination and subsequent p62-selective autophagic degradation of GPX4. Int J Biol Sci.

[42] Yang X, Zhu X, Sheng J, Fu Y, Nie D, You X (2024). RNF213 promotes Treg cell differentiation by facilitating K63-linked ubiquitination and nuclear translocation of FOXO1. Nat Commun.

[43] Ye W, Li Y, Wang W, Pan D, Wang X, Gu Y (2026). NEDD4 promotes reactive astrogliosis by enhancing K63-linked ubiquitination and inhibiting chaperone-mediated autophagy degradation of YAP1. Int J Biol Sci.

[44] Ferrari L, Bauer B, Qiu Y, Schuschnig M, Klotz S, Anrather D (2024). Tau fibrils evade autophagy by excessive p62 coating and TAX1BP1 exclusion. Sci Adv.

[45] Pugach EK, Richmond PA, Azofeifa JG, Dowell RD, Leinwand LA (2015). Prolonged Cre expression driven by the alpha-myosin heavy chain promoter can be cardiotoxic. J Mol Cell Cardiol.

