# Supplementary material for: Cardiomyocyte-Specific RNF128 Attenuates Pathological Cardiac Hypertrophy Progression by Stabilizing SERCA2a through Lys63-Linked Polyubiquitination
Source: Theranostics. 2026 Aug 12;16(15):8689–707. doi: 10.7150/thno.133221 (PMC13549231; doi:10.7150/thno.133221)
Supplement: Supplementary file 1 [file thnov16p8689s1.pdf]

Supplementary Materials

Cardiomyocyte-Specific RNF128 Attenuates Pathological Cardiac Hypertrophy Progression by Stabilizing SERCA2a through Lys63-Linked Polyubiquitination

Supplementary Figures

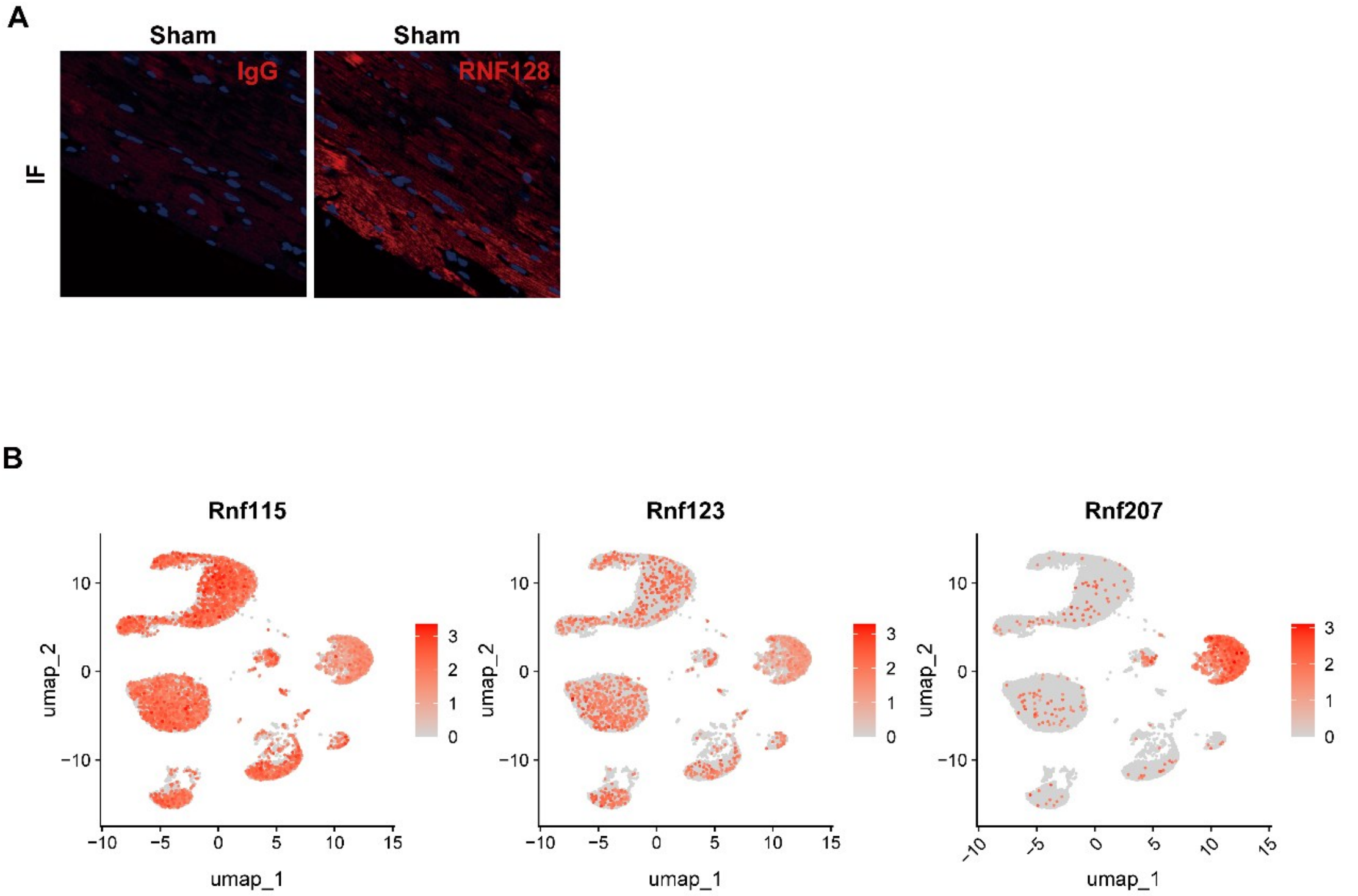

Supplementary Figure S1. RNF128 is downregulated in cardiomyocytes during cardiac hypertrophy.

A. Immunofluorescence of RNF128 and immunoglobulin G (IgG). **B**, Dot plots show cell-type specificity of *Rnf115*, *Rnf123* and *Rnf207*.

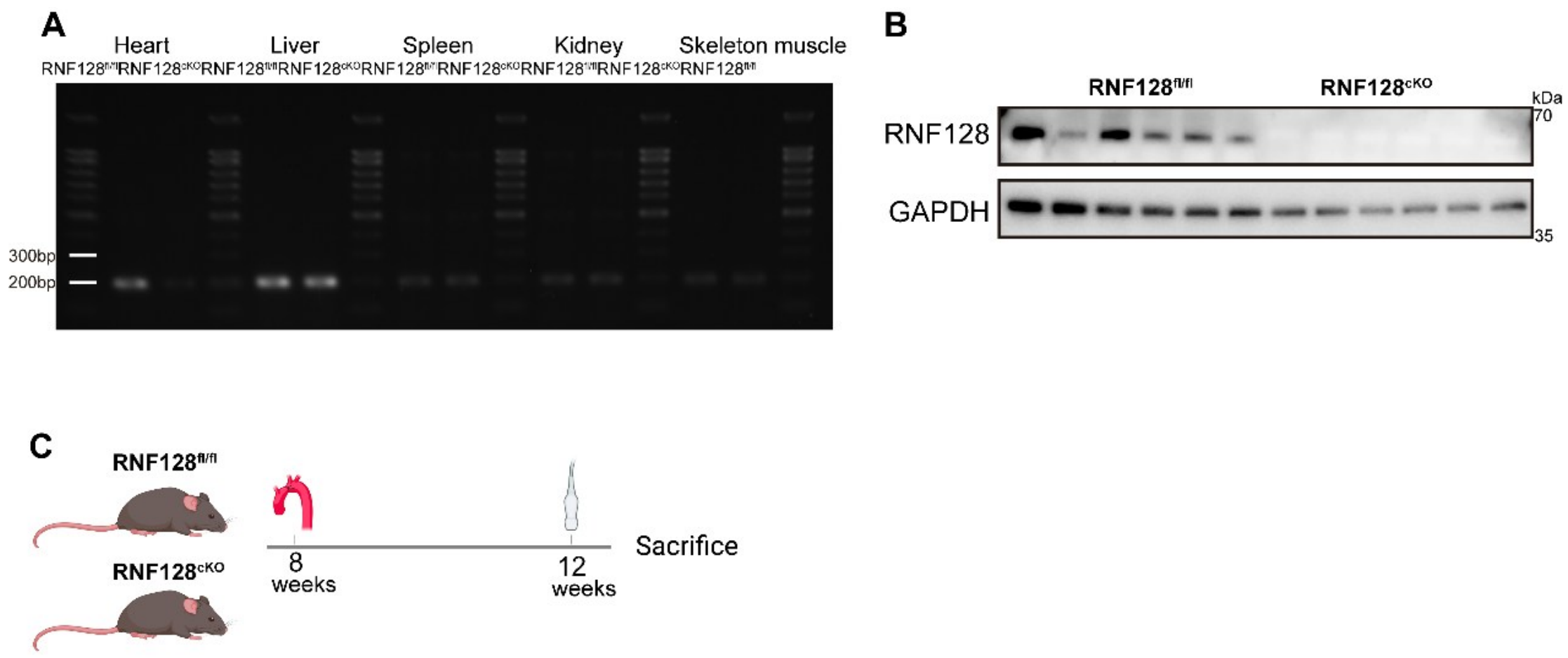

Supplementary Figure S2. RNF128 deficiency aggravates TAC (transverse aortic constriction)-induced cardiac hypertrophy.

A, Representative agarose gel electrophoresis of reverse transcription PCR products of several tissues. **B**, Representative immunoblotting of RNF128 from RNF128$^{fl/fl}$ and RNF128^{cKO} mice. **C**, Flowchart for TAC model.

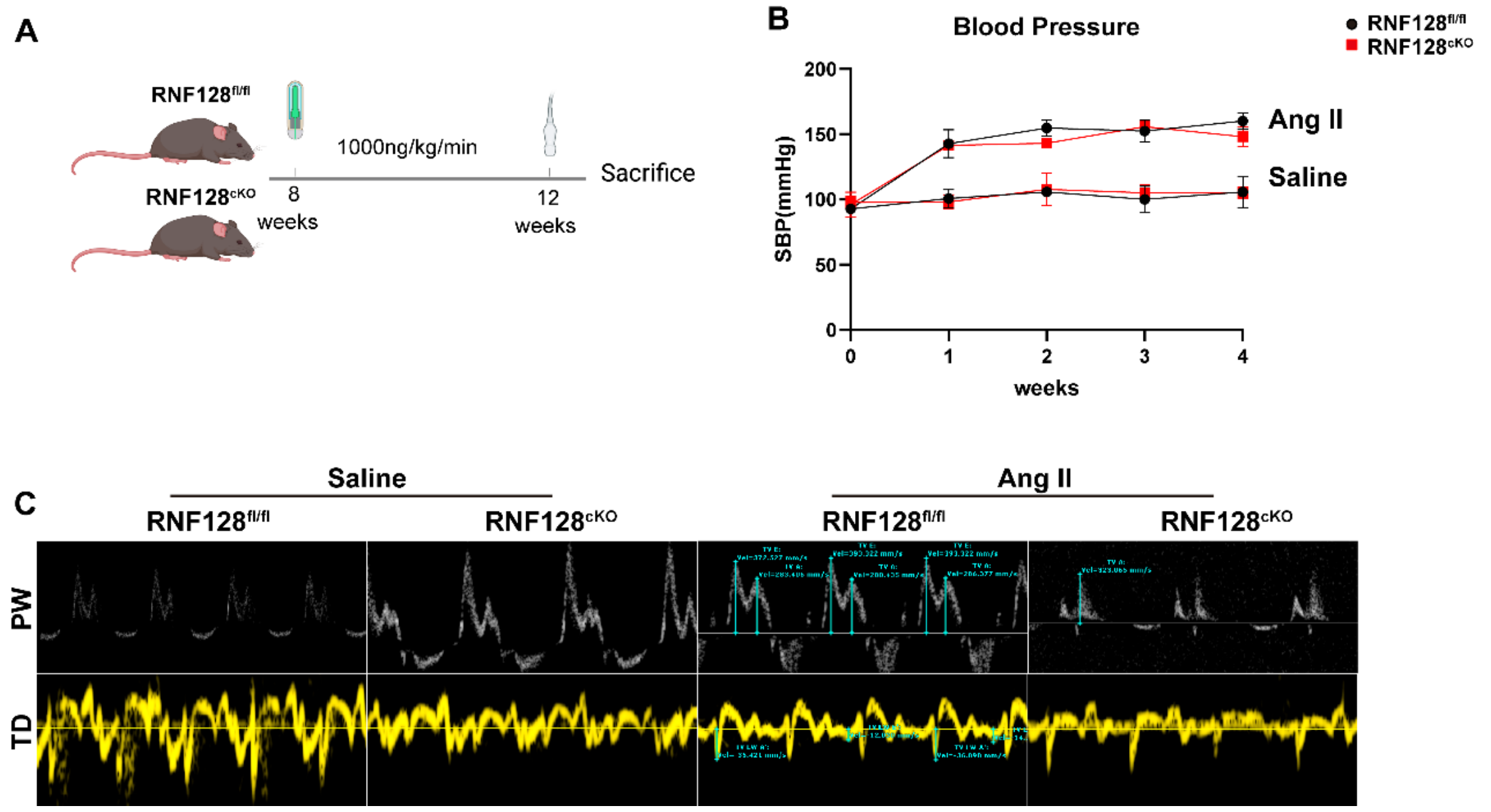

Supplementary Figure S3. RNF128 deficiency promotes angiotensin II (Ang II)-induced myocardial hypertrophy.

A, Flowchart for Ang II-infusion model. **B**, Blood pressure in the 4 groups in the Ang II-mediated model. **C**, Representative image of pulsed-wave tissue Doppler (PW) and tissue Doppler (TD).

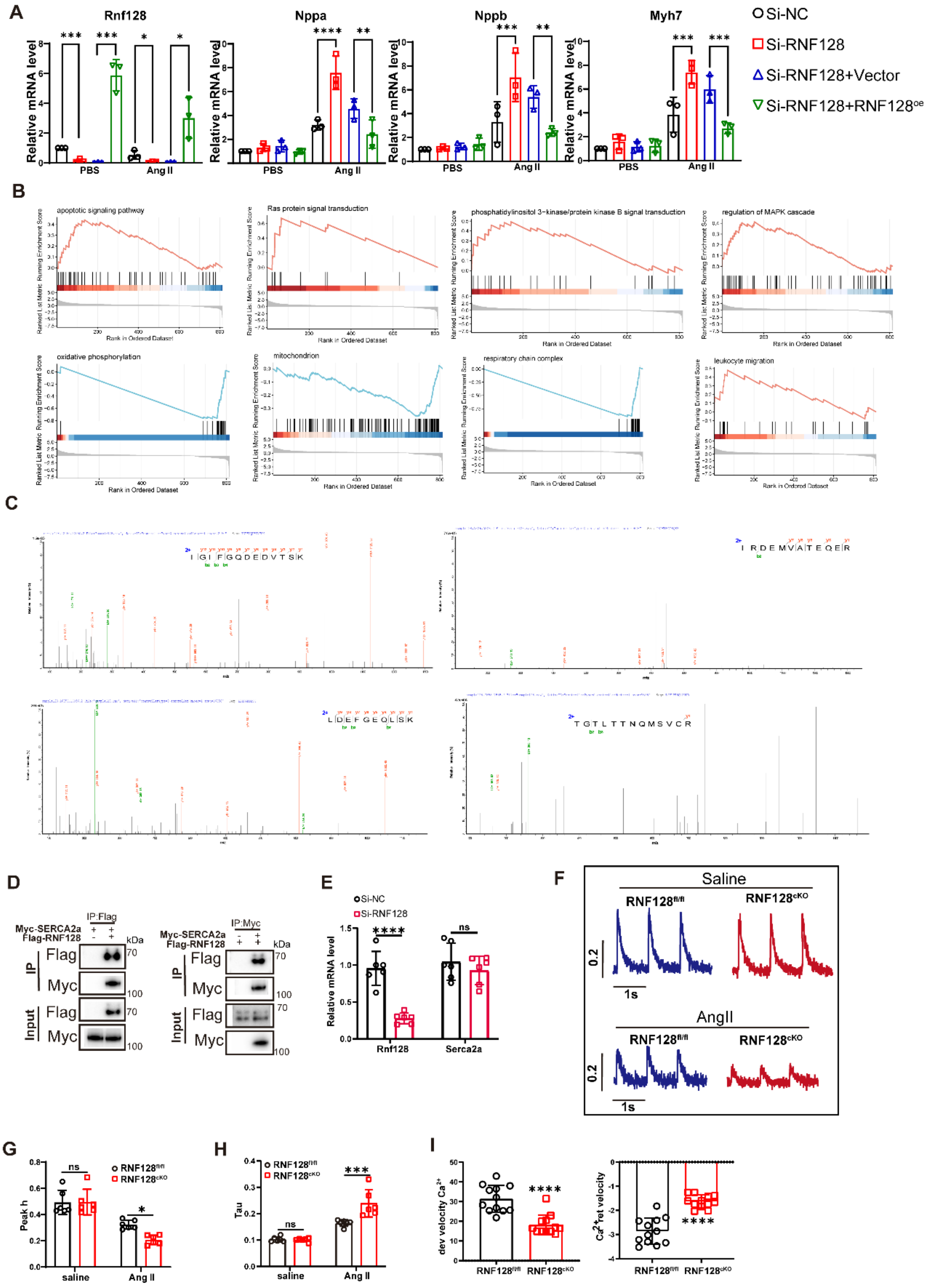

Supplementary Figure S4. RNF128 regulates cardiomyocyte hypertrophy, SERCA2a expression, and calcium handling.

A, Relative mRNA expression of *Rnf128*, *Nppa*, *Nppb* and *Myh7* in hypertrophic neonatal mouse cardiac myocyte (NMCM, 1 μM Ang II for 48 hours, n = 3 biological replicates, one-way ANOVA). **B**, GSEA (Gene Set Enrichment Analysis) of pathways in $RNF128^{cKO}$ mice. **C**, MS/MS spectra or representative SERCA2a peptides. Abbreviations for the amino acid residues are: A, Ala; D, Asp; E, Glu; F, Phe; G, Gly; I, Ile; K, Lys; L, Leu; M, Met; N, Asn; P, Pro; Q, Gln; R, Arg; S, Ser; T, Thr; V, Val; W, Trp. m/z, mass/charge ratio. **D**, Coimmunoprecipitation of RNF128 and SERCA2 in 293T cells. **E**, Relative mRNA expression of *Serca2a* and *Rnf128* in NMCM (n = 6 biological replicates, Student' s t-test). **F**, Representative calcium transient traces of adult mouse cardiomyocytes (AMCM). **G** to **H**, Quantitative detection of peak calcium transients and the Tau constant representing calcium recovery kinetics was performed using cardiomyocytes loaded with 2 μM Fura-2 AM fluorescence in adult mouse cardiomyocytes (AMCM). AMCMs were isolated from Ang II-treated $RNF128^{fl/fl}$ and $RNF128^{cKO}$ mice (n = 6 mice, one-way ANOVA). **I**, Departure velocity and the return velocity of calcium in AMCMs underwent Ang II treatment (n = 12 from 6 mice per group, Student's t-test).

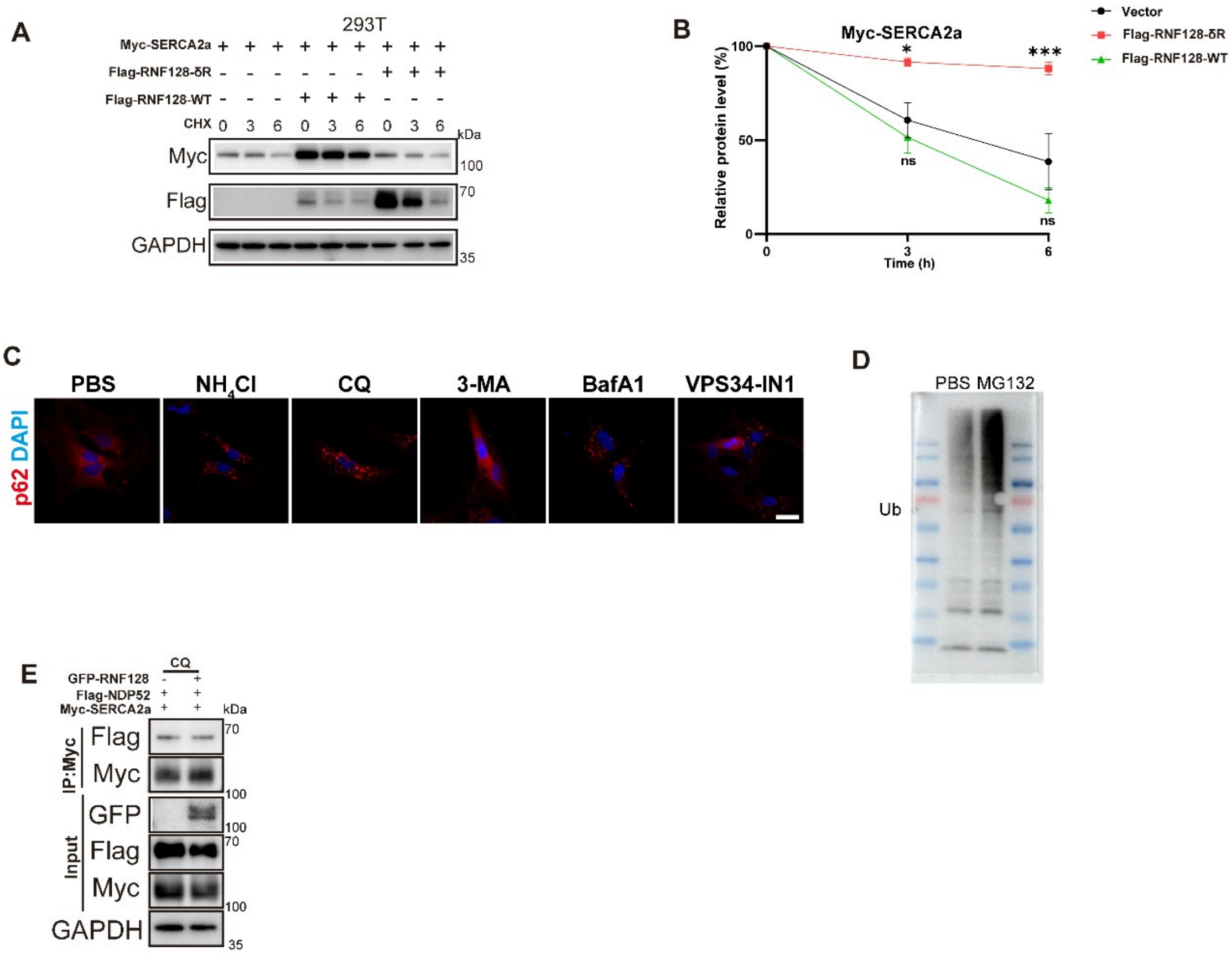

Supplementary Figure S5. RNF128 prevents SERCA2a's degradation.

A to **B**, Representative immunoblotting and densitometric quantification for Myc-SERCA2a in 293T subjected to cycloheximide (CHX, 50 μM) pulse-chase assay (n = 3 biological replicates, values were presented as mean ± SEM., two-way ANOVA followed with Šidák multiple comparisons test. Comparisons were made to Vector of each time). **C**, Immunofluorescence staining of SQSTM1/p62 in neonatal mouse cardiac myocyte (NMCM) treated with Chloroquine (CQ), Ammonium chloride (NH4Cl), and 3-Methyladenine (3-MA), bafilomycin (BafA1), VPS34-IN1 and Epoxomicin for 6 hours. **D**, Total ubiquitin detected in NMCMs treated with MG132 (carbobenzoxy-L-leucyl-L-leucyl-L-leucinal) for 6 hours. **E**, Representative western blotting of coimmunoprecipitation of SERCA2a with Flag-tagged NDP52 in 293T.

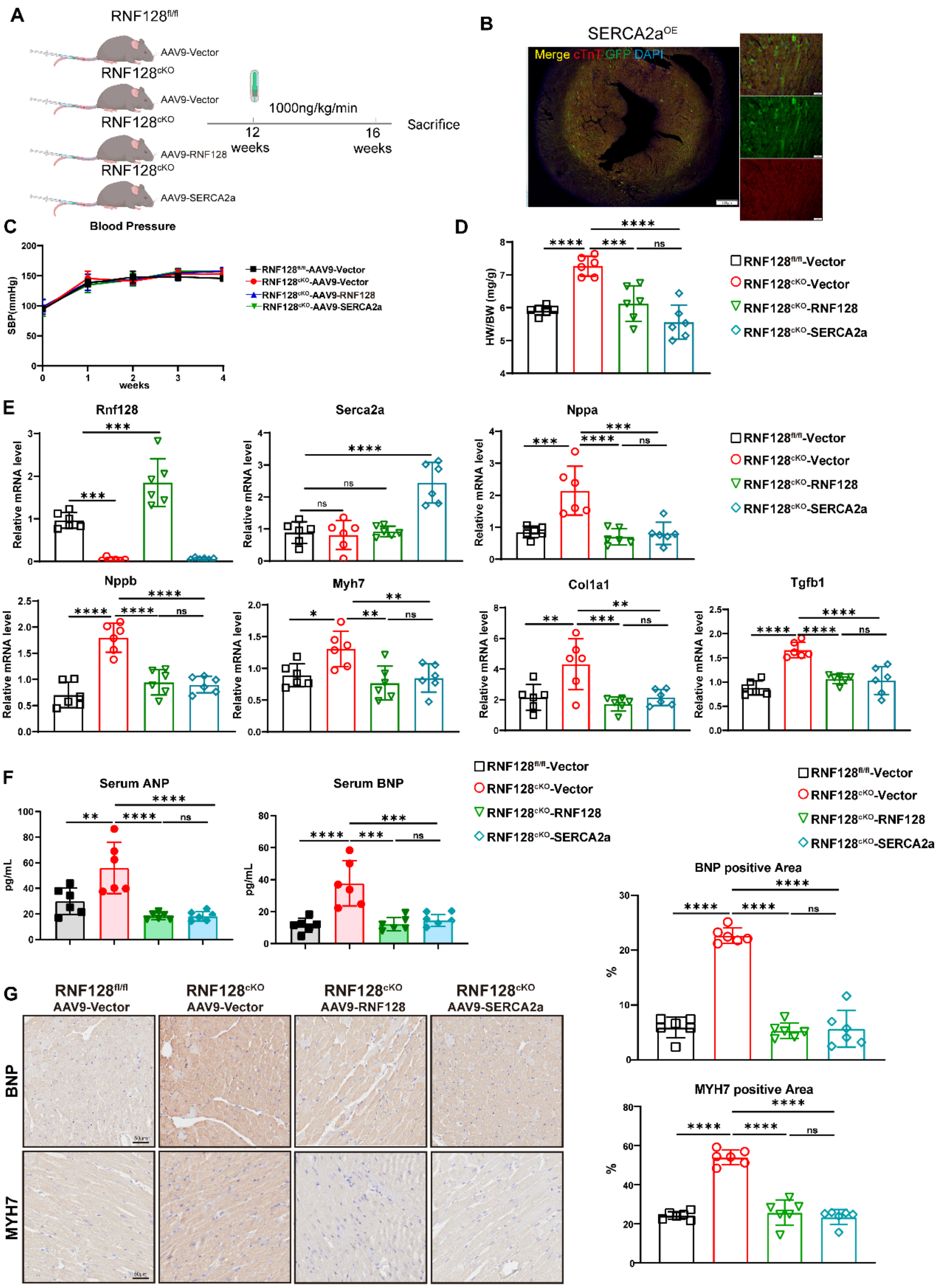

Supplementary Figure S6. RNF128 rescues cardiac dysfunction and pathological remodeling through SERCA2a in vivo.

A, Flowchart for restoration of RNF128 and SERCA2a in AngII model. **B**, Immunofluorescence staining of GFP in cTnT-AAV9-SERCA2a heart section. **C**, Blood pressure in the 4 groups. **D**, heart weight/body weight of 4 groups (HW/BW, n = 6 mice, one-way ANOVA). **E**, qRT-PCR analysis of *Rnf128, Serca2a, Nppa, Nppb, Myh7, Tgfb1 and Col1a1* in heart tissues. **F**, The serum ANP and BNP concentrations. **G**, IHC staining of BNP and MYH7 in heart sections. (**D**, **E**, **F**, **G**, n = 6 mice, one-way ANOVA with Dunnett's post-hoc test was used for intergroup comparisons. mRNA abundance in each experimental group was normalized to the $RNF128^{fl/fl}$ +AAV9-Vector defined as 1.).

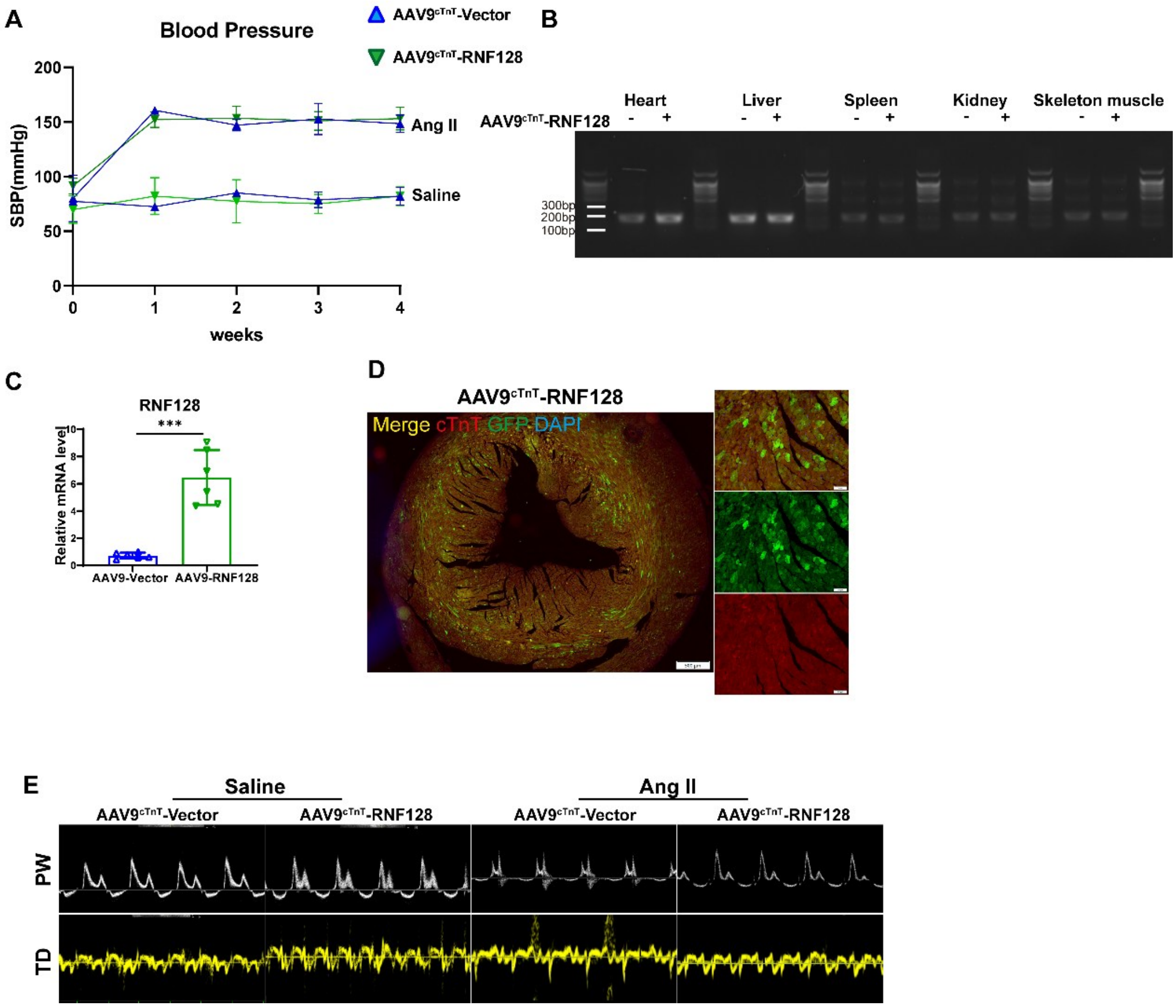

Supplementary Figure S7. RNF128 overexpression ameliorates Ang II-induced myocardial hypertrophy.

A, Blood pressure of 4 groups. **B**, Representative agarose gel showing RT-PCR products across different tissues. **C**, Relative mRNA expression of *Rnf128* in cTnT-AAV9-RNF128 heart (n = 6 mice per group, Student's t-test). **D**, Immunofluorescence staining of GFP in cTnT-AAV9-RNF128 heart section. **E**, Representative image of pulsed-wave tissue Doppler (PW) and tissue Doppler (TD).

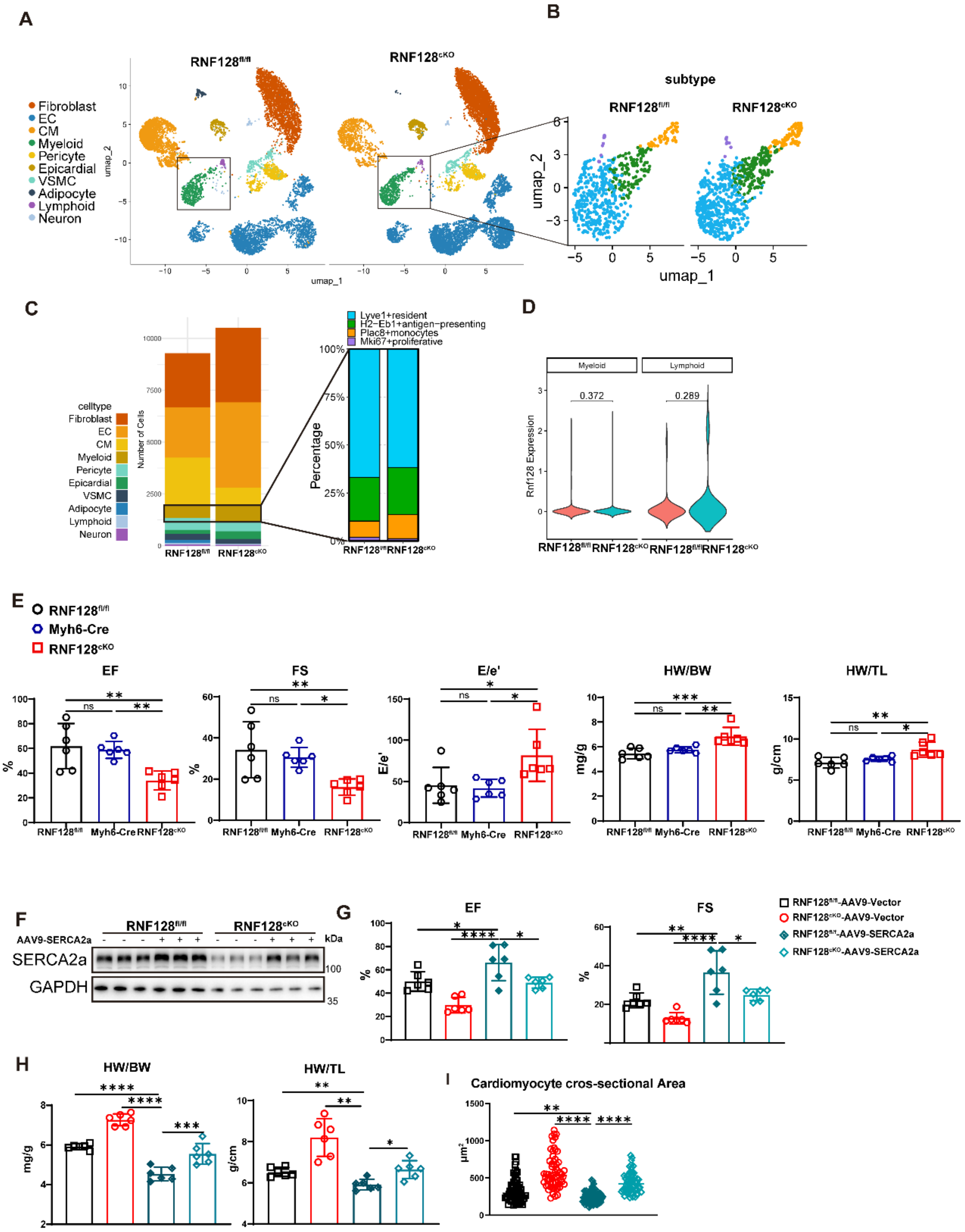

Supplementary Figure S8. Alterations of cardiac immune cell populations in RNF128$^{fl/fl}$ and RNF128^{cKO} hearts. Cardiac functional and morphological indexes of Myh6-Cre control mice and AAV9-SERCA2a-injected RNF128$^{fl/fl}$ mice.

A, UMAPs visualization of 10 cell types in TAC treated $RNF128^{fl/fl}$ and $RNF128^{cKO}$ mice. **B**, subtype of myeloid cells in $RNF128^{fl/fl}$ and $RNF128^{cKO}$ mice. **C**, percentage of myeloid-cell subtypes. **D**, RNF128 expression in myeloid and lymphoid cells. **E**, no significant differences in cardiac function and heart weight are observed between *Myh6-Cre* mice and $RNF128^{fl/fl}$ mice, whereas significant differences are detected compared with $RNF128^{cKO}$ mice. **F**, injection of AAV9-SERCA2a at a titer of 1×10^{11} vg/mouse restores SERCA2a expression in $RNF128^{cKO}$ hearts to the baseline level of $RNF128^{fl/fl}$. With the same viral titer, the total SERCA2a protein level in $\mathrm{RNF128}^{\mathrm{fl/fl}}$ cardiac tissues is higher than that in $RNF128^{cKO}$ cardiac tissues. **G** to **I**, overexpression of SERCA2a in $\mathrm{RNF128}^{\mathrm{fl/fl}}$ mice exerts a better rescue effect on cardiac function and cardiomyocytes hypertrophy than in $\mathrm{RNF128}^{\mathrm{cKO}}$ mice (the data presented in $\mathrm{RNF128}^{\mathrm{fl/fl}}$+ AAV9-Vector, $\mathrm{RNF128}^{\mathrm{cKO}}$+ AAV9-Vector and $\mathrm{RNF128}^{\mathrm{cKO}}$+ AAV9-SERCA2a are consistent with those described in the main text). (**E**, **G**, **I**, n = 6 mice per group, bar graphs represent mean ± SD, one-way ANOVA with Dunnett's post-hoc test, adjusted p-values displayed for multiple testing.)

Supplementary Table S1

Biometric and echocardiographic parameters in TAC-challenged mouse experiment

TAC model	Sham		TAC	
	RNF128$^{fl/fl}$	RNF128^{cKO}	RNF128$^{fl/fl}$	RNF128^{cKO}
	n = 6	n = 6	n = 6	n = 6
Heart rate (bpm)	416±10	412±8^{ns}	432±11^{ns}	435±25^{NS}
LVPW; s (mm)	1.13±0.09	1.09±0.09^{ns}	1.32±0.03*	1.58±0.16$^{###}$
LVPW; d (mm)	0.86±0.06	0.92±0.05^{ns}	1.12±0.04**	1.32±0.04$^{#}$
IVS; s (mm)	0.96±0.10	0.89±0.12^{ns}	1.17±0.09**	1.33±0.09$^{#}$
IVS; d (mm)	0.84±0.06	0.81±0.08^{ns}	1.00±0.06**	1.17±0.09$^{##}$
E/e’	23.39±11.81	21.56±9.19^{ns}	32.01±7.02	55.89±11.14$^{###}$
E/A	1.15±0.21	1.30±0.35^{ns}	1.45±0.10*	2.38±0.61$^{##}$
HW/BW, (mg/g)	4.54±0.16	4.32±0.45^{ns}	5.32±0.25*	6.42±0.84$^{##}$

ns, represents p > 0.05 vs RNF128$^{fl/fl}$ -Sham, * p < 0.05, ** p < 0.01, *** p < 0.001, **** p < 0.0001; NS, represents p > 0.05 vs RNF128$^{fl/fl}$ +TAC group, # p < 0.05, ## p < 0.01, ### p < 0.001, #### p < 0.0001.

Supplementary Table S2

Biometric and echocardiographic parameters in Ang II-challenged mouse experiment

Ang II model	Saline		Ang II	
(1000ng/kg/min)	RNF128$^{fl/fl}$	RNF128^{cKO}	RNF128$^{fl/fl}$	RNF128^{cKO}
	n = 6	n = 6	n = 6	n = 6
Heart rate (bpm)	449±14	416±10^{ns}	445±23^{ns}	429±35^{NS}
LVPW; s (mm)	1.11±0.05	1.09±0.16^{ns}	1.34±0.11*	1.59±0.20$^{\#}$
LVPW; d (mm)	0.85±0.09	0.89±0.08^{ns}	1.15±0.077***	1.27±0.22^{NS}
IVS; d (mm)	0.97±0.05	0.98±0.15 ^{ns}	1.18±0.08^{ns}	1.42±0.33^{NS}
IVS; s (mm)	1.19±0.14	1.13±0.12^{ns}	1.38±0.18	1.49±0.25
E/e’	23.44±3.55	35.89±10.88*	39.86±7.13**	52.39±5.76$^{\#}$
E/A	1.28±0.05	1.52±0.05***	1.80±0.12*	2.33±0.48$^{\#\#\#}$
HW/BW, (mg/g)	4.77±0.11	4.90±0.46 ^{ns}	5.97±0.32*	7.54±1.46$^{\#\#}$

ns, represents $p > 0.05$ vs RNF128$^{fl/fl}$ + Saline group, * $p < 0.05$, ** $p < 0.01$, *** $p < 0.001$, **** $p < 0.0001$; NS, represents $p > 0.05$ vs RNF128$^{fl/fl}$ + Ang II group, # $p < 0.05$, ## $p < 0.01$, ### $p < 0.001$, #### $p < 0.0001$.

Supplementary Table S3

Biometric and echocardiographic parameters in Ang II-challenged mouse experiment

Ang II model	Ang II			
	$RNF128^{fl/fl}$ +AAV9-VECTOR n = 6	$RNF128^{cKO}$ +AAV9-VECTOR n = 6	$RNF128^{cKO}$ +AAV9-RNF128 n = 6	$RNF128^{cKO}$ +AAV9-Serca2a n = 6
Heart rate (bpm)	428±12 ^{ns}	433±31	444±31^{ns}	432±27 ^{ns}
LVPW; d (mm)	1.04±0.07****	1.25±0.04	0.92±0.05****	1.10±0.09***
LVPW; s (mm)	1.50±0.23 ^{ns}	1.36±0.26	1.42±0.33 ^{ns}	1.21±0.10 ^{ns}
IVS; s (mm)	1.33±0.22 ^{ns}	1.48±0.11	1.32±0.15 ^{ns}	1.33±0.15 ^{ns}
IVS; d (mm)	0.93±0.05****	1.22±0.09	0.97±0.08****	1.01±0.10***
E/e’	30.95±9.39**	50.70±7.44	35.51±9.15*	29.10±10.21**
HW/BW (mg/g)	5.92±0.14***	7.27±0.30	6.13±0.54**	5.56±0.52****

ns, represents p > 0.05 vs $RNF128^{cKO}$+AAV9-VECTOR group, * p < 0.05, ** p < 0.01, *** p < 0.001, **** p < 0.0001.

Supplementary Table S4

Biometric and echocardiographic parameters in Ang II-challenged mouse experiment

Ang II model	Saline		Ang II	
	AAV9-VECTOR	AAV9-RNF128	AAV9-VECTOR	AAV9-RNF128
	n = 6	n = 6	n = 6	n = 6
Heart rate (bpm)	436±47	418±56^{ns}	426±21	439±20^{NS}
LVPW; s (mm)	1.12±0.15	1.15±0.18^{ns}	1.68±0.16	1.42±0.10$^{\#}$
LVPW; d (mm)	0.84±0.15	0.90±0.11^{ns}	1.21±0.07	1.05±0.04$^{\#}$
IVS; s (mm)	1.14±0.09	1.17±0.13^{ns}	1.68±0.16	1.42±0.07$^{\#\#}$
IVS; d (mm)	0.82±0.058	0.80±0.13^{ns}	1.08±0.09	0.92±0.06$^{\#\#}$
E/e’	31.00±5.44	28.10±5.89^{ns}	44.03±4.53	33.76±8.31$^{\#}$
E/A	1.16±0.23	1.24±0.06^{ns}	1.96±0.35	1.44±0.14$^{\#\#}$
HW/BW (mg/g)	4.69±0.17	4.53±0.16^{ns}	6.05±0.68	5.12±0.28$^{\#\#\#}$

ns, represents $p > 0.05$ vs AAV9-VECTOR + Saline group, * $p < 0.05$, ** $p < 0.01$, *** $p < 0.001$, **** $p < 0.0001$; NS, represents $p > 0.05$ vs AAV9-VECTOR + Ang II group, # $p < 0.05$, ## $p < 0.01$, ### $p < 0.001$ #### $p < 0.0001$.

Supplementary Table S5

MS/MS spectra peptide sequences and scores of SERCA2a from RNF128-IP sample.

**Sequence**	**Protein**	**Score**
LDEFGEQLSK	SERCA2a	147.2
IGIFGQDEDVTSK	SERCA2a	112.15
TGTLTTNQMSVCR	SERCA2a	107.24
IRDEMVATEQER	SERCA2a	105.46
HTDPVPDPR	SERCA2a	95.815
DIVPGDIVEIAVGDK	SERCA2a	90.731
KSEIGIAMGSGTAVAK	SERCA2a	72.705
TASEMVLADDNFSTIVAAVEEGR	SERCA2a	65.901
TVEEVLGHFGVNESTGLSLEQVK	SERCA2a	32.952

Abbreviations for the amino acid residues: A, Ala; C, Cys; D, Asp; E, Glu; F, Phe; G, Gly; H, His; I, Ile; K, Lys; L, Leu; M, Met; N, Asn; P, Pro; Q, Gln; R, Arg; S, Ser; T, Thr; V, Val; W, Trp; Y, Tyr.

Supplementary Table S6

Clinical information of human samples.

	Sex	Age (years)	IVS (mm)	EF
Hypertrophic-1	female	59	16	0.68
Hypertrophic -2	male	71	12	0.68
Hypertrophic -3	male	61	19	0.55
Hypertrophic -4	female	76	14	0.7
Hypertrophic -5	male	66	13	0.5
	Sex	Age(years)	LVID (mm)	EF
DCM-1	female	59	64	0.1
DCM-2	male	40	83	0.31
DCM-3	male	62	74	0.18
DCM-4	male	34	69	0.12
DCM-5	female	65	82	0.19
	Sex	Age(years)	Clinical diagnosis	
NCC-1	male	30	SAH	
NCC-2	male	34	Brainstem hemorrhage	
NCC-3	male	46	Brainstem hemorrhage	
NCC-4	male	33	Brainstem hemorrhage	

DCM, dilated cardiomyopathy. NCC, non-cardiac disease control. SAH, subarachnoid hemorrhage.

Methods

Echocardiography

In this study, mice anesthetized with 1.5% isoflurane and examined using a high-frequency ultrasound system (Vevo 3100, FUJIFILM Visual Sonics, Canada). The mice were positioned in the supine position, and parasternal long-axis views were first obtained to delineate cardiac anatomy. Subsequently, M-mode imaging was performed at the mid-papillary muscle level to measure left ventricular end-diastolic diameter (LVID; D), end-systolic diameter (LVID; S), and wall thickness, with ejection fraction calculated accordingly. From the apical four-chamber view, pulsed-wave (PW) Doppler was applied at the mitral valve tips to capture the left ventricular inflow spectrum, analyzing peak velocities of the E-wave and A-wave, as well as the E/A ratio. Simultaneously, tissue Doppler imaging was utilized at the lateral mitral valve to measure early diastolic velocity (e'), with the E/e' ratio calculated to evaluate left ventricular function. All data acquisition was performed under synchronized ECG monitoring and respiratory gating. Post-processing analysis was conducted using dedicated software, averaging data from three consecutive cardiac cycles to ensure the stability and reproducibility of the measurements.

Cell Culture and Transfection

The HEK293T cells were purchased from OriCell Therapeutics (Cyagen Bioscience Inc., Guangzhou, China). Cell culture medium: 4.5 g/L glucose DMEM medium +10% fetal bovine serum +1% penicillin/streptomycin. Cells were maintained in a humidified incubator at 37°C with 5% CO_2. Transfection was performed when cell confluence in the 6-cm dish reached

80%. 3 μg plasmid was transfected in Opti-MEM™ Medium containing 3 μL Lipofectamine™3000 and 3 μL P3000.

The isolation of neonatal mouse cardiomyocytes was performed as follows: Briefly, 3-day-mice were euthanized. Hearts were excised and rinsed with PBS. The cardiac tissue was then digested using 0.125% EDTA-trypsin in 4 ℃ overnight. Tissues were digested with 1 mg/mL collagenase type II at 37 °C for 10 min per cycle. The cell-containing supernatant was harvested and centrifuged after each incubation, fresh collagenase was added to the residual tissue pellet, and the digestion cycle was repeated four times. Separation of cardiomyocytes from fibroblasts was performed by differential adherent culture for 2h. Isolated cardiomyocytes were cultured in medium: 4.5 g/L glucose DMEM medium +10% new-born bovine serum +5-BrdU. After 48 hours of culture, the cardiomyocytes exhibited regular rhythmic contractions, confirming their suitability for subsequent experiments. To evaluate the role of RNF128 in cardiac hypertrophy, NMCMs were treated with 1 μM angiotensin II.

Adult mouse cardiomyocyte isolation was performed according to a published protocol[1]. Detailed steps are as follows: EDTA buffer rinse the right ventricle. Hearts were excised following cardiac arrest. The aorta was ligated to a 27G needle. The EDTA buffer was then replaced with perfusion buffer. Collagenase was then injected into the left ventricle and complete digestion was achieved. The cardiac tissue was thoroughly minced with forceps and agitated for 1 minute, after which digestion was terminated using stop buffer. Isolated cardiomyocytes were filtered through 100 μm mesh to centrifuge tubes, and purified via gravity sedimentation (20 minutes each time, repeated three times) with calcium-containing buffer. The

resulting pellet was resuspended in culture medium. Viable AMCMs were utilized in subsequent experiments.

Intracellular Calcium Ion Detection

NMCMs were loaded with 2 μM Fluo-4 AM and incubated at 37 °C for 20 min. After the probe was loaded, cells were washed with PBS thrice. Primary cardiomyocytes were incubated in DMEM medium and placed in a spinning-disk confocal microscope (Andor, Oxford) to detect the intracellular calcium transient.

Measurement of cardiomyocyte shortening and Ca^{2+} transient

Electrically stimulated adult mouse cardiomyocytes (AMCMs) were subjected to steady-state conditioning via 1 Hz pacing, followed by synchronized contractile activity monitoring. Real-time sarcomere dynamics were captured using the IonOptix imaging platform (IonOptix Co., MA, USA). Contractile function was quantified as fractional shortening [$(DeltaL/L_{diastolic}) \times 100\%$], where ΔL represents systolic-diastolic length differential. Data were acquired from n = 12 cells per experimental cohort to ensure statistical robustness.

Calcium dynamics in adult mouse cardiomyocytes (AMCMs) were assessed using Fura-2 AM (2 μM), a Ca^{2+}-sensitive fluorescent probe. Cells were incubated with Fura-2AM for 30 min at 37°C under continuous Tyrode's buffer perfusion. The MyoPacer EP system (IonOptix, MA, USA) was applied to deliver 1 Hz electrical pacing. Fluorescence signals reflecting Ca^{2+} transients were acquired using an IonOptix calcium imaging platform, with subsequent quantification performed through IDL analytical software (Research Systems Inc.).

Western Blotting and Co-IP

Cardiac tissue and primary cardiomyocyte proteins were isolated using CelLytic™ M lysis reagent. Protein samples of equal concentration were loaded onto 10% SDS-PAGE gels and then transferred to PVDF membranes. 5% bovine serum albumin (BSA) was used for membrane blocking for 1 hour at room temperature. Primary antibody incubation was at 4°C for 12–16 hours. Membranes were probed with HRP-linked secondary antibodies (1:5000 dilution) for 1 hour after TBST washes. Protein signals were visualized using enhanced chemiluminescent (ECL) substrate and captured with a chemiluminescence imaging system. Band grayscale intensity was analyzed using ImageJ software. The relative expression of each protein was calculated as the ration of target-band intensity to control band intensity from the same sample. All quantitative results were generated from at least three independent biological replicates.

Protein Co-IP was performed according to the standalone antibodies or antibody-conjugated agarose beads protocols. Briefly, cells were harvested and lysed in 0.5 ml RIPA buffer + protease inhibitors. Lysate was sonicated for 10 s, and clarified by 13,000 rpm centrifugation for 15 min, 4°C. 400 μL of the cleared lysate was mixed with either primary antibodies or antibody-conjugated agarose beads for immunoprecipitation and subjected to overnight incubation at 4°C. The bead-antibody-protein complexes were pelleted via centrifugation (speed and duration as specified in the protocol), followed by five rigorous washing cycles with RIPA buffer to remove nonspecific interactions. The complexes were then resuspended in Tris-glycine SDS sample buffer (2×), boiled at 99°C for 10 min, and centrifuged to collect the

supernatant for downstream immunoblotting analysis.

TRITC-Phalloidin Staining

For F-actin labeling, we carried out TRITC-phalloidin staining in accordance with standard experimental procedures. Primary cardiomyocytes were seeded on cell slides, subjected to two rounds of preheated sterile PBS rinsing, and fixed with 4% formaldehyde at room temperature for 5 min. Specimens were thoroughly permeabilized by repetitive washing with 0.1% Triton X-100 PBS buffer. Next, incubation in dark with TRITC-modified phalloidin reagent was performed for 30 min. DAPI-supplemented mounting medium was used for nuclear counterstaining prior to microscopic fluorescence imaging and image acquisition.

Real-time quantitative PCR

We isolated and purified total RNA from myocardial tissue using the Trizol isolation system. The Fastgen reagent was utilized to enrich intact mRNA from cultured cardiomyocytes. The generation of cDNA was accomplished by reverse transcription employing the Takara PrimeScript™ RT reagent Kit (DRR037A, Takara, Japan). SYBR Green-based quantitative polymerase chain reaction was conducted with the designated cycling parameters: 10 minutes of initial denaturation at 95°C, and 35 iterative cycles of 15 seconds at 95°C and 45 seconds at 60°C. Custom oligonucleotide primers were obtained from BGI Genomics.

ELISA

The contents of ANP (Research Cloud Biology, Shandong, China) and BNP (Research Cloud Biology, Shandong, China) in mice serum were measured according to the reagent instructions.

Adeno-Associated Virus Infection

To overexpress RNF128 or SERCA2a in vivo, adeno-associated virus serotype 9 (AAV-9) encoded RNF128 (AAV9-RNF128) and SERCA2a (AAV9-SERCA2a) or Vector (AAV9-Vector) were injected into mice. AAV-9 was purchased from WZ Biosciences Inc. (Shandong, China). As shown in Figure S6 and Figure 8, *C57BL/6* mice and $RNF128^{fl/fl}$ / $RNF128^{cKO}$ mice were divided into corresponding groups (n = 6). AAV9 was injected via tail vein (1×10^{11} vg/mouse) four weeks before hypertrophy model. Upon finishing cardiac function assessment, mice were euthanized to collect blood and heart tissue specimens for subsequent assays.

Histological Analysis

The cardiac tissue was immersed in 4% formaldehyde for fixation, followed by a standard dehydration and clearing process before being embedded in paraffin blocks. Using a microtome, 5-μm-thick sections were prepared for subsequent histological analyses. The tissue slices were then stained with Hematoxylin and Eosin (H&E) (Product #G1120, Solarbio Life Sciences, Beijing, China). Paraffin tissue sections were sent to Wuhan Servicebio Technology Co., Ltd. for Masson’s trichrome and Picrosirius Red staining. Stained sections were delivered back to our lab for imaging and quantitative measurement. Wheat Germ Agglutinin staining was performed following the respective manufacturers' protocols.

Immunofluorescence staining

Paraffin-embedded tissue sections were baked at 65 °C for 2 h before deparaffinization. Sections were washed three times with phosphate-buffered saline (PBS). Antigen retrieval was

performed by heating slides to 95 °C in sodium citrate buffer for 15 min, followed by natural cooling to room temperature. Sections were blocked with 5% donkey serum for 30 min at room temperature to reduce non-specific binding. Primary antibodies were diluted to optimized working concentrations and incubated with sections at 4 °C overnight. After three PBS washes, fluorophore-conjugated secondary antibodies were applied and incubated for 2 h at room temperature. Slides were mounted using DAPI-containing antifade mounting medium and scanned with a digital whole-slide scanner. Immunofluorescence images were captured using either an Olympus digital pathology slide scanner or a Zeiss laser scanning confocal microscope. Identical image acquisition settings were used for all samples in each independent experiment. At least six random, non-overlapping fields were imaged per biological sample. Quantitative analysis was conducted using ImageJ software to calculate the percentage of positively stained area within each standardized field of view.

RNAscope

RNAscope kit was purchased from Advanced Cell Diagnostics, Inc. According to the protocol, sections were first baked, then deparaffinized by xylene and rehydrated through graded ethanol. Followed by hydrogen peroxide treatment, high-temperature target retrieval, and protease digestion to expose nucleic acids and antigens. RNA probe hybridization and cascade signal amplification were performed, with target RNAs labeled using TSA/Opal fluorophores. Immunofluorescence staining was subsequently completed via incubation with HRP-conjugated secondary antibodies, and the sections were finally stained with DAPI, mounted with antifade medium, and subjected to fluorescence imaging. Quantification was performed

using ImageJ software. RNA Scope signals were quantified by counting the positive dots per view.

Materials

Antibody	Cat No.	Supplier and	Application
Flag	F1804	Sigma	WB, 1:1000
Myc	AE070	Abclonal	WB, 1:1000
HA	AE105	Abclonal	WB, 1:1000
Gapdh	2118	CST	WB, 1:1000
SERCA2	ab150435	Abcam	WB, 1:4000
Phospho-CaMKII	12716	CST	WB, 1:1000
CaMKII	A0198	Abclonal	WB, 1:1000
RNF128	-	Abclonal	WB, 1:500; IF:1:50
Myh7	MABT849	Merck	WB,1:1000;IHC,1:200
ANP	ab225844	Abcam	WB, 1:1000
BNP	A23996	Abclonal	WB,1:1000;IHC:200
Collagen I	ab260043	Abcam	WB, 1:1000
ATG5	12994	Cell Signaling Technology	WB, 1:1000
ATG7	8558	Cell Signaling Technology	WB, 1:1000
SQSTM1/p62	610832	BD Transduction Laboratories™	WB, 1:1000
NDP52	A7358	Abclonal	WB, 1:1000
LAMP2	ab13524	Abcam	IF: 1:200
cTnT	ab8295	Abcam	IF: 1:500

Reagent	Cat No.	Company
Fura-2AM	S1052	Beyotime Biotechnology
Fluo-4AM	S1060	Beyotime Biotechnology
cTnT-AAV9(RNF128^{oe}, SERCA2a^{oe} and Empty Vector)	-	WZ-Biosciences Inc
Opti-MEM^{TM} Medium	51985034	Gibco
LipofectamineTM3000 P3000	L3000-015	Thermo Fisher Scientific
LipofectamineTMRNAiMAX	13778150	Thermo Fisher Scientific
5-BrdU	B5002	Sigma Aldrich
Collagenase II	LS004176	Worthington
CelLytic™ M	C2978-250ML	Sigma Aldrich
TGX Stain-Free™ FastCast™ Acrylamide Kit, 10%	1610183	Bio-Rad
TRITC-labeled Phalloidin	RM02835	Abclonal
DAPI	ab104139	Abcam
Carbobenzoxy-L-leucyl-L-leucyl-L-leucinal (MG132)	HY-13259	MCE, DMSO
Chloroquine (CQ)	HY-17589A	MCE, DMSO
Ammonium chloride (NH4Cl)	HY-Y1269	MCE, H2O
3-Methyladenine (3-MA)	HY-19312	MCE, H2O

cycloheximide (CHX)	HY-12320	MCE, DMSO
293T (HEK-293T)	H4-1401	OriCell Therapeutics (2022.Mar.22)
Osmotic Pump	2004W	RWD
ELISA kit ANP	KYY-0590M1	Research Cloud Biotechnology
ELISA kit BNP	KYY-0060M1	Research Cloud Biotechnology

**Primers**	**Forward**	**Reverse**
Mouse-NPPA	AAGAACCTGCTAGACCACCTGGAG	TGCTTCCTCAGTCTGCTCACTCAG
Mouse-NPPB	CCAGTCTCCAGAACAATCCA	GCTTGAACTATGTGCCATCTT
Mouse-MYH7	CAGAACACCAGCCTCATCAACCAG	CACCTTGCGGAACTTGGA
Mouse-COL1A1	TGGCCTTGGAGGAAACTTTG	CTGTAGGTGAAGCGACTG
Mouse-Tgfb1	CCAGATCCTGTCCAAACTAAGG	GCCAGGAATTGTTGCTGTATT
Mouse-βACTIN	CTACCTCATGAAGATCCTGACC	CACAGCTTCTCTTTGATGTCAC
Mouse-SERCA2a	TTCTGCTTATCTTGGTAGCCAA	CTTTCTGTCCTGTCGATACACT
Mouse-RNF128	GGCAGTTAAAGGCAGATG	GGCAAGTCCTGTGTTCTA
**Si-RNA**	**Sense**	**Anti-sense**
Si-RNF128-mouse	GGUCAUCGAAGUAGGGAAATT	UUUCCCUACUUCGAUGACCTT
Si-SERCA2a-mouse	CUGCUAACUUCAUCAAAUATT	UAUUUGAUGAAGUUAGCAGTT
